# Categorizing and assessing comprehensive drivers of provider behavior for optimizing quality of health care

**DOI:** 10.1371/journal.pone.0214922

**Published:** 2019-04-17

**Authors:** Elisabeth Engl, Steve Kretschmer, Mokshada Jain, Saransh Sharma, Ram Prasad, B. M. Ramesh, Mrunal Shetye, Seema Tandon, Sanjiv Kumar, Tisa Barrios Wilson, Sema K. Sgaier

**Affiliations:** 1 Surgo Foundation, Seattle, Washington, United States of America; 2 Final Mile Consulting, Mumbai, India; 3 Final Mile Consulting, Chicago, Illinois, United States of America; 4 Department of Community Health Sciences, University of Manitoba, Winnipeg, Canada; 5 Bill & Melinda Gates Foundation, New Delhi, India; 6 India Health Action Trust (IHAT), Uttar Pradesh Technical Support Unit (TSU), Lucknow, India; 7 Department of Global Health & Population, Harvard T.H. Chan School of Public Health, Boston, Massachusetts, United States of America; 8 Department of Global Health, University of Washington, Seattle, Washington, United States of America; BITS Pilani, INDIA

## Abstract

Inadequate quality of care in healthcare facilities is one of the primary causes of patient mortality in low- and middle-income countries, and understanding the behavior of healthcare providers is key to addressing it. Much of the existing research concentrates on improving resource-focused issues, such as staffing or training, but these interventions do not fully close the gaps in quality of care. By contrast, there is a lack of knowledge regarding the full contextual and internal drivers–such as social norms, beliefs, and emotions–that influence the clinical behaviors of healthcare providers. We aimed to provide two conceptual frameworks to identify such drivers, and investigate them in a facility setting where inadequate quality of care is pronounced. Using immersion interviews and a novel decision-making game incorporating concepts from behavioral science, we systematically and qualitatively identified an extensive set of contextual and internal behavioral drivers in staff nurses working in reproductive, maternal, newborn, and child health (RMNCH) in government public health facilities in Uttar Pradesh, India. We found that the nurses operate in an environment of stress, blame, and lack of control, which appears to influence their perception of their role as often significantly different from the RMNCH program’s perspective. That context influences their perceptions of risk for themselves and for their patients, as well as self-efficacy beliefs, which could lead to avoidance of responsibility, or incorrect care. A limitation of the study is its use of only qualitative methods, which provide depth, rather than prevalence estimates of findings. This exploratory study identified previously under-researched contextual and internal drivers influencing the care-related behavior of staff nurses in public facilities in Uttar Pradesh. We recommend four types of interventions to close the gap between actual and target behaviors: structural improvements, systemic changes, community-level shifts, and interventions within healthcare facilities.

## Introduction

Improving health outcomes by enhancing quality of care within healthcare facilities has become a key goal of healthcare programs in low- and middle-income countries, as inadequate quality of care is one of the primary causes of patient mortality [1, 2]. Since provider behaviors are a critical component of quality of care, understanding why healthcare providers behave as they do is key to designing effective interventions to address the gaps in care quality. In this paper, we outline how staff behavior can be systematically investigated, using a case study from government public health facilities in Uttar Pradesh, India’s most populous state. In the study, we focused on outcomes in reproductive, maternal, newborn, and child health (RMNCH), as this is a priority area for the state and a key focus of the health-related United Nations Sustainable Development Goals. Maternal and neonatal mortality rates in Uttar Pradesh are still among India’s highest [3, 4]. Neonatal mortality has remained stagnant for a decade despite an increase in institutional deliveries, indicating that quality of care in facilities is sub-optimal [3, 5]. While many components of the health system, such as households behaviors, care provided by informal providers, and effectiveness of front-line workers are likely to influence mortality, adverse RMNCH outcomes are disproportionately concentrated within facilities: over half of all child mortality in Uttar Pradesh is neonatal mortality, and 75% of that is early neonatal mortality [6]. Maternal mortality has declined over the last decade, but at 285 per 100,000 live births is still among the highest in India [4]. The quality of care that healthcare providers deliver must therefore be a key focus of improving RMNCH outcomes.

The public healthcare system in Uttar Pradesh, as in the rest of India, is tiered. Subcenters and primary health centers focus on the lowest—village cluster—level, followed by community health centers (CHC) and 24/7 primary health centers (PHC) at the block level, and finally district hospitals (DH). District hospitals are headed by a chief medical surgeon, who may have other medical officers to provide specialist support. At the block level, medical officers in-charge lead facilities, supported by medical officers or lady medical officers. Staff nurses are present at all facilities, except at the subcenter level, where the auxiliary nurse midwives provide services. The type of maternal and child care offered also differs between facilities. For example, subcenters and primary health centers can manage simple deliveries, but complications and complex pregnancies are referred to higher-level centers such as community health centers and district hospitals. Importantly, the theoretical structure can differ in practice due to staff shortages. For example, as many positions remain vacant, auxiliary nurse midwives can be found working at higher centers than their designated facility.

Large-scale efforts to strengthen RMNCH care at the facility level have focused on improving the skills, knowledge and practices of service providers, and removing supply-side barriers to care. Interventions have included investments in infrastructure and supply chains, increasing the number of facility staff and improving human-resources processes, providing staff training, and creating new roles such as nurse mentors at the block level. Nurse mentors are dedicated to supporting staff nurses in their labor, delivery and immediate postpartum services by providing skills demonstrations, on-site mentoring, and helping them adhere to guidelines and clinical practice. Evaluations of nurse mentors in other states found that care competence of staff nurses has indeed increased [7, 8]. A recent quantitative survey [9] showed that solving supply issues and other external gaps is indeed a critical prerequisite for optimal quality of care in facilities. Nurses in well-equipped facilities adhered to labor management practices better, and providing case sheets and skills training by nurse mentors increased the clinical knowledge, skills, and guideline adherence of staff [9]. At the same time, these interventions are unlikely to completely optimize quality of care, as significant gaps in both clinical skills and routine practices remained. For example, only 5% of nurses were able to demonstrate the complete set of steps for Active Management of the Third Stage of Labor (AMTSL) in the first survey, and this increased to an improved, but still low, 22% in the follow-up period. In their practice, no nurses measured all critical vital signs of deliveries initially, and only 5% did so in the second round. In other clinical behaviors, more pronounced improvements were measured, but even there, deficiencies remained. For example, nurse assistance with initiation of breastfeeding increased from 27% to 65% between the surveys, and weighing of the newborn from 50% to 77% [9]. Overall, there are substantial gaps between knowledge and skills and applied practice: “know-do” gaps where knowledge and skills fail to result in behavior, and conversely “do-know” gaps where behaviors are carried out, but with incomplete knowledge or skills. Without further insights into what drives these still sub-optimal behaviors, the RMNCH program in Uttar Pradesh is implementing prescriptive management tools, but cannot identify and implement transformative behavior-change levers effectively throughout the system.

Globally, there is a wealth of studies on how nursing quality of care and associated health outcomes are affected by structural factors within healthcare, such as staffing levels and support systems [10–12]. Similar to the quantitative survey mentioned above, these studies generally find that improving structural problems can reduce, but not eliminate, negative health outcomes in hospitals. For instance, in a large study in developed countries, nurse reports of low quality of care were three times more likely in hospitals with lower nurse staffing and support levels [11], but increasing staffing levels did not consistently result in decreased mortality or complication levels [13]. In addition, factors such as staff experience, access to knowledge resources, and the opportunity to engage in teamwork also influence quality of care [14]. Recently, a striking trial of a coaching-based implementation of the WHO Safe Childbirth Checklist in Uttar Pradesh showed that knowledge- or training-focused interventions do not always lead to better outcomes [15]. The study found no significant effect of increased checklist use on maternal and perinatal health outcomes, despite healthcare staff adhering to guidelines more than in control facilities (with varying levels of change across behaviors). However, checklist use and adherence to practices was not sustained after coaching ceased. The authors speculated that this may be due to “lack of checklist stock, staff belief that they knew the items on the checklist, lack of enthusiasm, or other reasons”. [15] While each practice outlined in the checklist has a clear link to mortality, and the precise reasons for the lack in a change in outcomes are unclear, this trial highlights that simply providing a checklist and being trained to use it is not enough.

While research into the influence of external processes on quality of care is relatively well established, an understanding of internal, cognitive factors that might impact nurse practices, such as in the trial using the checklist, is severely lacking. Where it is available, research is often of low quality, as most data is obtained via self-report questionnaires directly probing for reports on motivations or emotions. One exception to the lack of data is research on stress: while there is little research in India, globally, stress is frequently reported as a significant problem for nurses. The available evidence suggests that much of this is driven by excessive workloads, pressures of work-life balance, and the emotional demands of healthcare work [16–19]. Coping strategies for reducing stress can include avoidance [20], and over-confident attitudes to work [21], as well as planned problem-solving, self-control, and seeking social support [22]. However, research into other cognitive influences on behavior, such as risk perception, motivation, cognitive biases, or self-efficacy (the belief in one’s ability to accomplish a task), is very limited. This is surprising, given that there are many indications that cognitive biases, such as attribution bias, play a role in clinical practices. For example, when nurses and other healthcare workers in the United Kingdom were asked about the causes of MRSA infections, they generally attributed infections to the behaviors of other people and situational factors, such as patients, but attributed successes in infection control to good team performance [23]. As another example, when looking to make evidence-based decisions, doctors’ and nurses’ prior beliefs affect the answers they find when searching for evidence in literature [24]. These kinds of confirmation and attribution biases may impact the quality of clinical decision-making. Overall, research on how internal drivers of behavior impact the quality of care that nurses provide is limited, relies primarily on direct self-report, and comes predominantly from developed countries.

**Fig 1:**
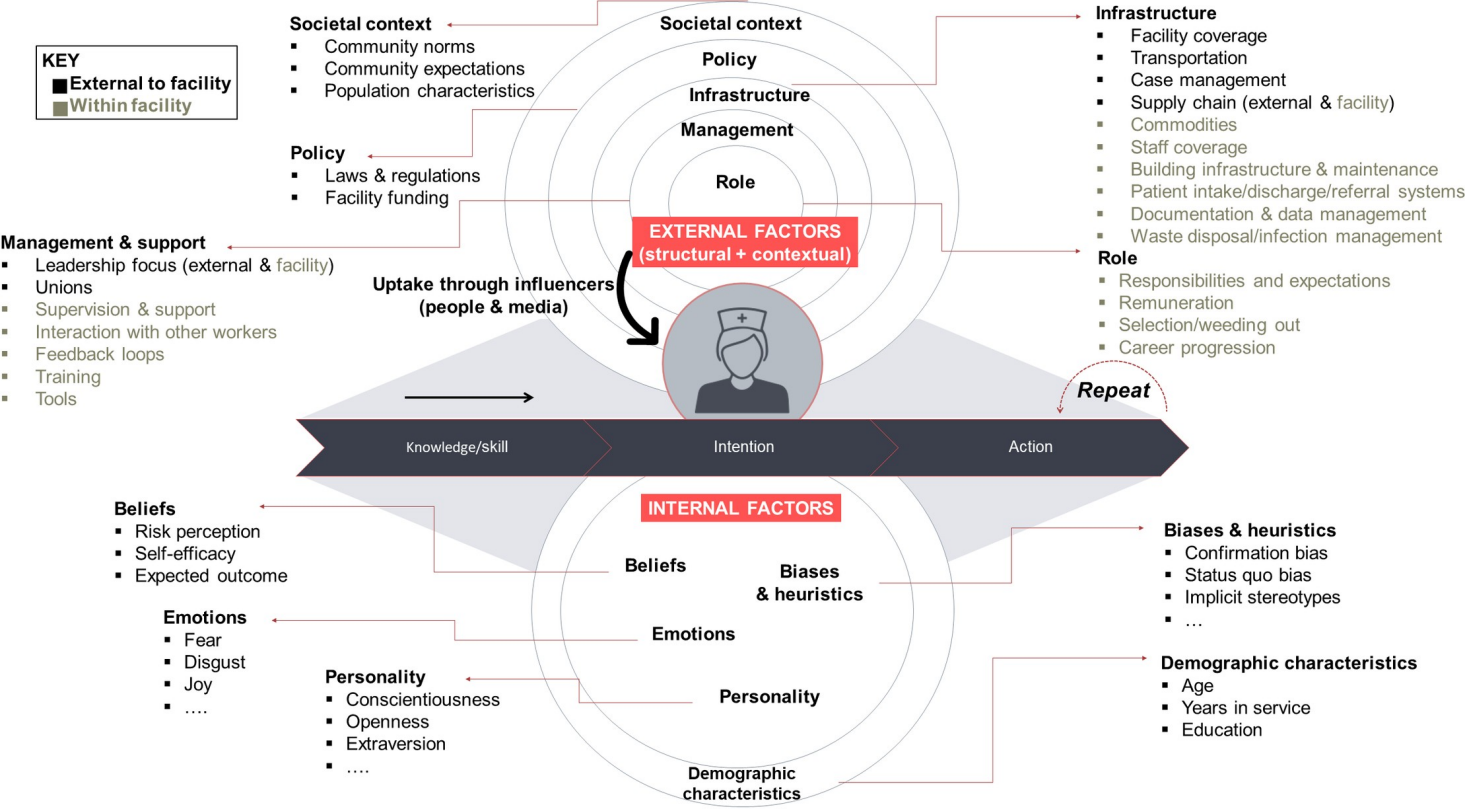
Framework for Unpacking Provider Practices (UPP). The framework outlines the complex set of factors influencing facility outcomes via the clinical practices of staff.

**Fig 2:**
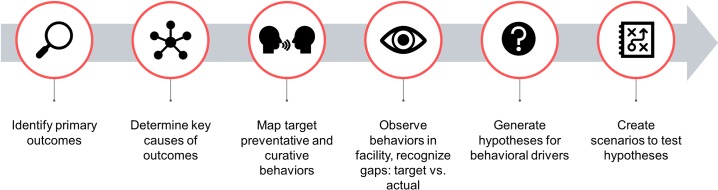
Pipeline showing the main components of the Providers Outcomes Pathway (POP) to develop interview guides and scenarios for the decision-making game. For the full Providers Outcomes Pathway used, see S1 Fig.

**Fig 3:**
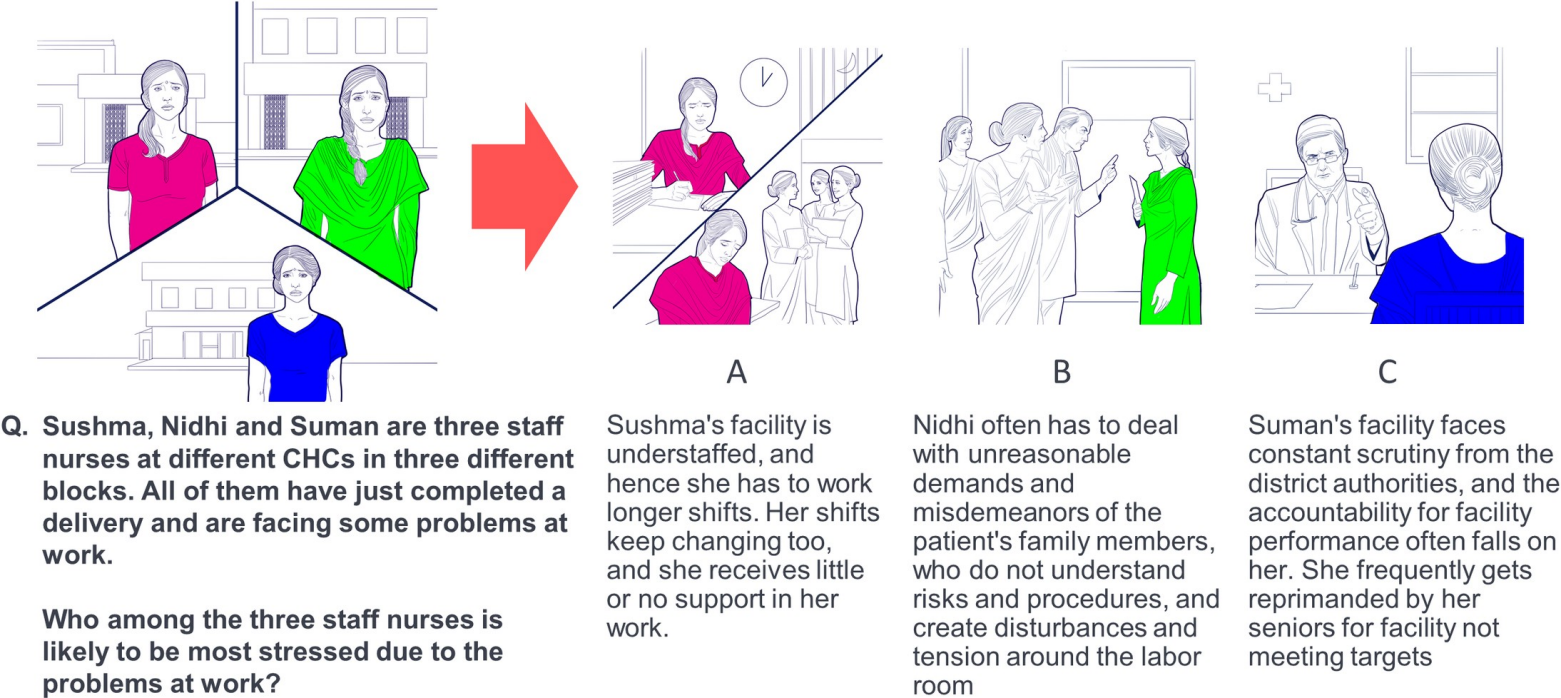
Sample decision-making game scenario. The text was played via speaker to participants, who selected one answer. This scenario investigated the focus areas ‘Stress and coping mechanisms’ and ‘Reluctance of patients’ (see Fig 4).

**Fig 4:**
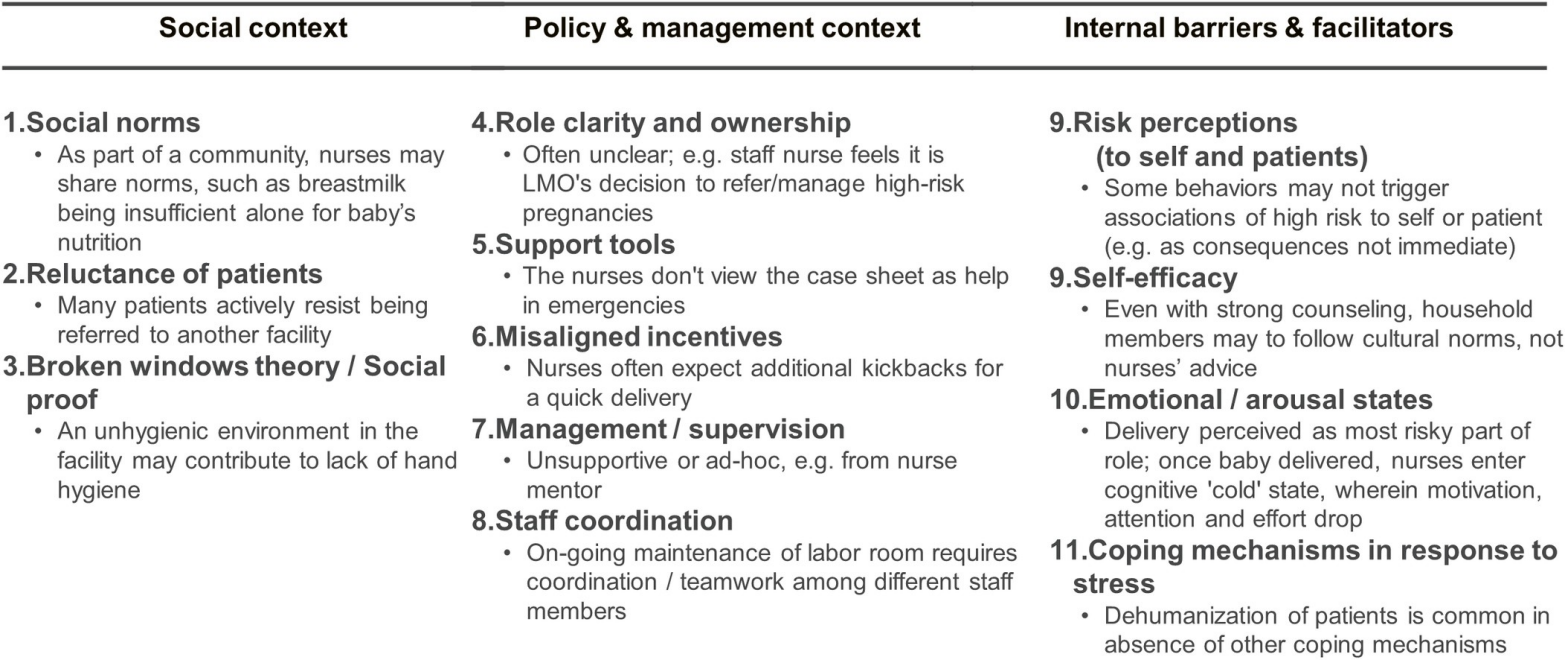
The 12 focus areas identified as likely to drive critical behaviors of staff nurses in facilities. These 12 factors were selected out of 17 found in total, based on the frequency with which they occurred in facility-based immersion interviews.

**Fig 5:**
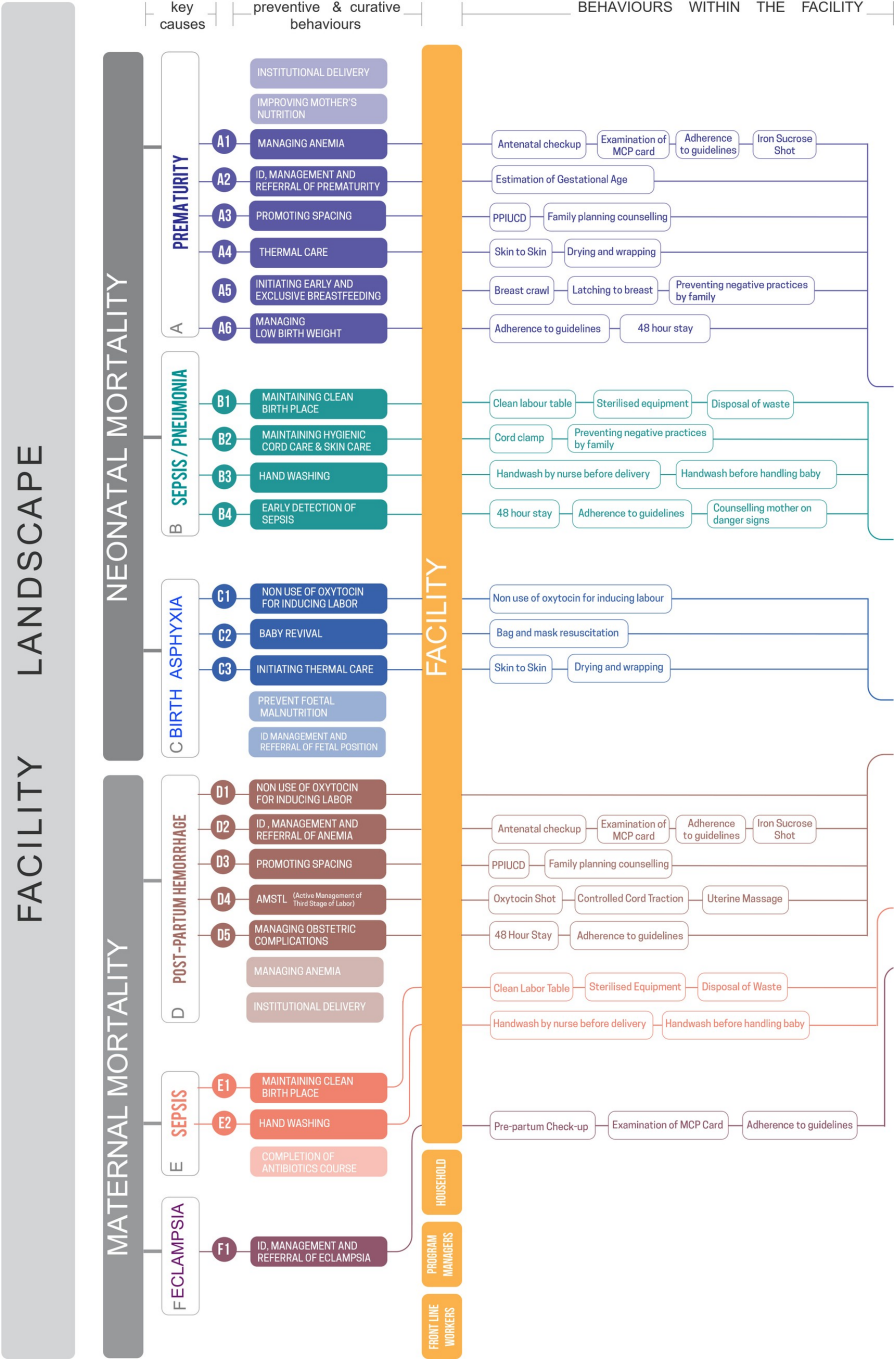
Excerpt from the Providers Outcomes Pathway (POP), showing the pathway from key causes of maternal and neonatal mortality to behaviors within the facility. As next steps, these behaviors were compared to insight from immersions, which were then linked to focus areas examined in detail in the decision-making game (see S1 Fig for the full pathway).

**Fig 6:**
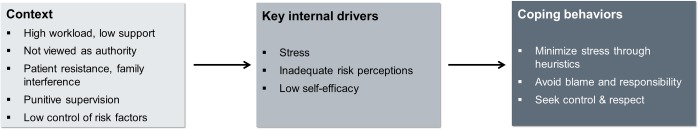
Contextual influences on staff nurse drivers of behavior and associated coping behaviors, as distilled from immersions and responses to decision-making game scenarios.

**Fig 7:**
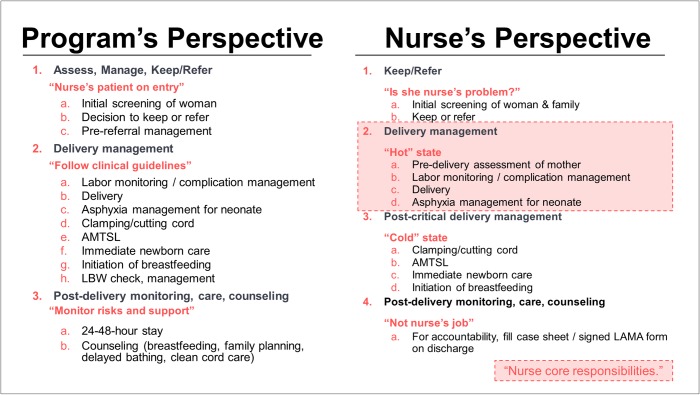
Program and staff perception of the nurse role. Programs and staff nurses perceive the role of the nurse differently, resulting in a gap between program expectations and actual behaviors.

**Fig 8:**
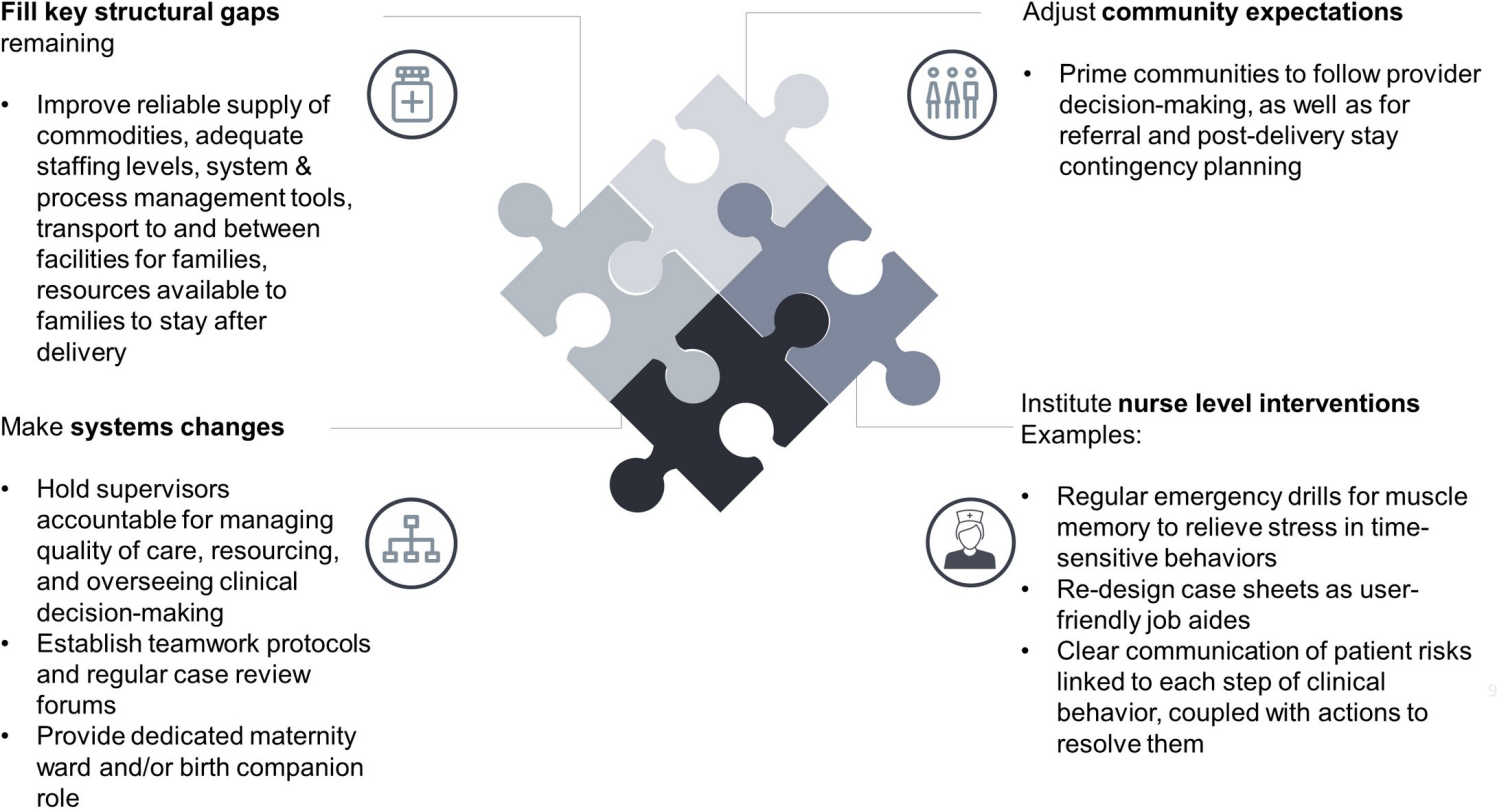
Summary of suggested recommendations and priorities to improve nurse quality of care within facilities.

This study has several objectives. First, we introduce a theory-driven process for systematically structuring and refining the types of potential drivers and barriers driving key healthcare staff behaviors (Figs 1–5). Second, we introduce a set of qualitative methods–facility-based ‘immersion’ interviews and a novel decision-making game (Fig 3)—to investigate these drivers and barriers among healthcare staff in public health facilities in Uttar Pradesh, India. We focus especially on under-studied internal drivers and barriers. Third, we outline the holistic picture of contextual and internal drivers of behavior found along a series of behaviors along the maternal and neonatal health pathway (Figs 6 and 7). Finally, we discuss potential levels of intervention to address these multifaceted barriers to adequate quality of care (Fig 8). Some of these interventions are currently being adopted by the government of Uttar Pradesh.

## Materials and methods

Ethics approval was obtained from the Sigma Research and Consulting Institutional Review Board, New Delhi, India (IRB number 10020/IRB/D/16-17).

### Structuring drivers of behavior with the Framework for Unpacking Provider Practices (UPP)

The literature reviewed above had revealed a large set of factors at different levels driving facility outcomes as well as the clinical practices of staff. We structured these factors into a conceptual approach (Fig 1), the Framework for Unpacking Provider Practices (UPP). The components of the UPP framework are applicable to understanding healthcare provider behavior beyond the context in this study. For this study, the framework served as a guideline to structure the broad potential *drivers and barriers* to capture in initial qualitative research, and the coding of the resulting transcripts.

The many contextual influences on behavior can be internal to the individual facilities where providers work, or external to them. Beyond and/or within individual facilities, social factors as well as factors related to policy, infrastructure, management or specific role characteristics set the context in which facility staff operate. However, their clinical practices are also influenced by a set of internal drivers, which operate in complex interdependence among each other and the contextual environment. For a given behavior, these behavioral drivers—mediated by influencers—first lead to the healthcare provider becoming aware of a correct clinical behavior (i.e., by obtaining knowledge and skills), then forming an intention to carry out the behavior, and finally acting. This illustrates that awareness on its own does not necessarily lead to action. The components of the UPP framework may be generalized to understanding other types of healthcare providers, while the specific content for each component will be determined by the specific role investigated. Crucially, while provider characteristics such as knowledge and skills are readily tracked, underlying internal factors driving human decision-making are typically not measured. On a broad level, these internal factors, which we focused on in this study, include beliefs (such as risk perceptions, or beliefs about self-efficacy, i.e., the ability to control outcomes), emotions, and unconscious biases that facilitate some behaviors and inhibit others. Knowledge of the correct practices and the skills to implement are often measured, but internal drivers that are critical to build their intention to perform the target behavior, which is a key predictor of actual behaviors, are often ignored [25].

### Providers Outcomes Pathway (POP) from outcomes to likely behavioral drivers

Following from the general conceptual UPP framework, we developed a Providers Outcomes Pathway (POP) to determine which clinical *behaviors* to probe and focus on in the field work in order to shift target outcomes, generate hypotheses around the more specific drivers of behavior likely to be involved, and explore these drivers in more detail (Fig 2 and S1 Fig).

First, we considered the key maternal and neonatal causes of mortality: eclampsia, sepsis, and post-partum hemorrhage (maternal), and birth asphyxia, sepsis/pneumonia, and premature birth [26, 27]. We then listed the key clinical causes of each outcome and the preventive or corrective behavior that should be employed by facility staff to address each cause. In the case of post-partum hemorrhage, examples would be abstaining from oxytocin to induce labor, active management of third stage of labor, and promoting the spacing of children through family-planning counseling and inserting a post-partum intrauterine contraceptive device (PPIUCD). We then probed these key behaviors in the facility-based ‘immersion’ interviews (see below), from which we formed hypotheses on the likely causes of the gap between target and observed behavior. These hypotheses then formed the basis of the scenarios in a decision-making game developed to investigate in more detail which factors influenced what areas of decision-making.

### Facility-based immersion interviews

As a first step, in-depth ‘immersion’ interviews of healthcare staff, conducted within facilities during the workday, provided preliminary qualitative insights into the factors driving behaviors relevant to the target clinical outcomes. Specifically, the objectives of immersions were to prioritize staff roles within facilities that may have a predominant impact on quality of care for further study, observe staff behavior in the context of their daily work, and develop more targeted hypotheses for decision-making game scenarios.

#### Facility sampling and staff enrollment

The sampling process for the immersion interviews consisted of two stages: selecting the districts, blocks, and facilities from which staff were drawn, and selecting staff members for participation within facilities. Immersion interviews were conducted in a sample of 8 facilities, 6 community health centers (CHC), 1 district women’s hospital (DH), and 1 primary health center (PHC) in 4 districts in Uttar Pradesh. This approach was designed to reflect a variety of facility and community contexts.

First, districts were selected based on being within ~ a 3-hour radius from the state capital, Lucknow, as well as the presence of a Technical Support Unit (TSU) program in at least some blocks of each district. The TSU is tasked with supporting the state government to increase the efficiency, effectiveness and equity of the delivery of key RMNCH services, and was able to facilitate our access to facilities. Within each district, blocks were chosen to reflect a mixture of the following characteristics: whether blocks had a TSU program in place or not (and in the case of TSU programs, one block was chosen because TSU had implemented a Nurse Mentor program), and whether community literacy levels, the percentage of rural dwellers, and the percentage of community members coming from Scheduled Castes/Scheduled Tribes were high or low. To sample 6 CHC-type facilities, 6 different blocks in 4 districts were selected (each block contained one main CHC), of which 3 were TSU-operated (one of which had a Nurse Mentor program), and 3 were not. In addition, from the selected blocks the DH closest to Lucknow, and a PHC that was closest to the DH, were chosen (see S1 Table for details).

After facilities were selected, letters from district-level government officials were obtained to grant access to facilities to research staff. Research staff spent one unannounced day per facility conducting interviews with staff from a variety of roles present that day, who had not been briefed in advance. Selection was based on availability within the facility that day, and written staff consent was taken at the time of interview. The mixed-gender interview sample consisted of 7 staff nurses, 7 nurse mentors, 4 lady medical officers (LMOs), 2 medical officers, 2 medical officers in-charge, 1 chief medical superintendent, 6 pharmacists, 3 cleaning staff, 1 adolescent reproductive and sexual health counselor, 1 family planning counselor, and 1 labor-room helper. On average, 1–2 staff nurses and at most one person of each other role were present in a given shift on each day. All who were present and deemed available by facility management consented to be interviewed and completed the interview.

#### Immersion interview process

The interviewer team consisted of professional field interviewers who were native Hindi-speakers (see S2 Table for interviewer characteristics). Interviews were conducted individually and in private in the respective facilities, lasted for 30 minutes to 1 hour, and used semi-structured interview guides (see S1 File for the staff nurse discussion guide, which was used as a template for interviewing other facility-based roles). The research team also took informal field notes of staff attendance, facility condition, and clinical behaviors of staff members.

#### Immersion interview analysis

Audio recordings of the interviews were transcribed, then translated to English for analysis. The interviews were analyzed using thematic analysis [28]. Coding was conducted by a team of 4 behavioral scientists employed at Final Mile, and was done in MS Excel. Individual notes were compared, and discrepancies were resolved through qualitative sense-making and triangulation. Coding tags were closely tied to the UPP framework (Fig 1) and included—among others—beliefs, job satisfaction and interactions with other staff and patients, structural context of the facility, emotions, perceived norms, and training and other processes. Then, quotes corresponding to the areas of interest were extracted and summarized as findings. Transcripts or analyses were not returned to participants for comments or feedback. Following analysis, the coding tags were refined for the decision-making game qualitative analysis (see below). Field notes from observations were used to supplement available data and decide on the focus of decision-making game scenarios. As research staff had limited access within facilities and observations were carried out on one day per facility, clinical behaviors were not quantified, but are narratively summarized together with immersion interview analysis in S1 Fig.

### Ethnolab: A behavioral decision-making game

The immersion interviews and the Providers Outcomes Pathway from health outcomes to behavioral focus areas formed the basis of Ethnolab, an audio-visual decision-making game methodology developed by the behavior change company Final Mile.

#### Sampling

Following the immersion interviews, staff nurses were found to be involved in most of the clinical behaviors identified in the Providers Outcomes Pathway. Nurses were therefore chosen as the main focus for the subsequent decision-making game. The final sample consisted of 46 female staff nurses from a participating 39 CHCs, 2 DHs and 4 PHCs. The sampling process again following a two-step process of facility-, then staff selection. A total of 7 group game sessions were initially scheduled, and for each session, ~8 nurses from different facilities with the same facility performance level were invited to participate. 5 sessions were scheduled to be held with nurses from CHCs (3 TSU, 2 non TSU), and 1 session each with nurses from PHCs and DHs. For the facility selection process, facilities were classified into performance categories (high, average, low) based on a set of parameters representing facility readiness and selected health behaviors, as well as geographical considerations. Among others, parameters included outcomes of complication management, reporting of complications, availability of critical resources (equipment and drugs), available data on nurse training, skills and practices, and delivery load. Not all parameters were used for all types of facilities, given that different parameters are routinely collected (and applicable) for different facility types (see S2–S6 Files for extensive detail on parameters).

After facilities were chosen, information on all staff nurses employed in each facility was collected: age, type of contract (permanent or contractual), and years of work experience. The goal was to select a heterogeneous total sample of nurses, who could then be grouped relatively homogeneously within game session groups to facilitate a trusting environment in each session. On this basis, 1 or (in two instances) 2 nurses were invited to attend from each facility (see S3 Table for staff selection and characteristics).

For logistical reasons, the game in the DH could not be held. Out of 48 staff nurses initially selected and invited across other facility types, 30 attended (Table 1). Informal follow-up suggested travel time to the research facility and timing conflicts due to shift work as the main reasons for non-attendance. Participants had a mean age of 34.2 ± 9.8 (SD) years, and a wide variety of tenure between 1 and 30 years (mean = 6.7 ± 9.8 (SD) years). 80% of nurses were contractually employed, 20% were permanent employees. All nurses were female.

**Table 1:** Main facility and nurse sample for the decision-making game.

Group #	Facility type	Performance category	TSU/Non- TSU	Staff nurses attended/invited
1	CHC	High	TSU	4 / 8
2	CHC	Average	TSU	5 / 9
3	CHC	Low	TSU	8 / 8
4	CHC	High	Non-TSU	5 / 8
5	CHC	Low	Non-TSU	4 / 7
6	PHC	Average	TSU	4 / 8

30 out of 48 invited nurses completed the decision-making game. A booster sample of 16 nurses was then added.

Due to low attendance resulting from the original sample design, a booster sample of 3 adjoining TSU districts of Lucknow, which had not previously been included, was added. Each DH, and all 14 average-performing CHCs in the booster sample were selected. To maximize participation, facilities were asked to provide a staff nurse for the game based on availability. Out of 14 CHCs, 13 agreed to enroll, and a total of 14 staff nurses participated. Out of the 3 DHs, 2 staff nurses from 2 DHs participated. Demographic characteristics were not collected for the booster sample. In both the main and the booster sample, no participants dropped out during the course of data collection once at the location.

#### Decision-making game process

Written consent was obtained from all participants. Decision-making games were held in a central research location rather than in individual facilities. As for immersions, participants received no compensation, but in this case were compensated for travel to the research location.

The decision-making game flow was structured as follows: each session comprised a group of ~8 participants, previously clustered by similarity in age and tenure. Using a projector and speakers, participants were presented with an audio-visual narrative of a series of scenarios illustrating a health-related situation, featuring a protagonist similar in age, gender, and socio-economic background to the participants. No group was given all scenarios, but each scenario was included in at least one group. At the end of each scenario, participants were given a choice of three decisions the protagonist of the scenario could make (Fig 3).

Using a remote control, participants were instructed to vote for the option they thought the majority of other participants would choose. This was done in order to reduce social desirability bias. The game element of Ethnolab, designed to increase engagement, consisted of a scoring system, where the highest score was awarded to the person who most often guessed the majority decision correctly. After the game, ‘hot-state’ interviews (meaning the perception and reactions to the scenarios were still fresh in the participants’ mind) were conducted with participants broken into small groups to further delve into their responses (see S8 File for the discussion guide).

Seventeen decision-making scenarios were developed (see S7 File for full scenario texts), which aimed to cover all main clinical behaviors and interaction with other clinical staff in a realistic way. Each scenario could test for the involvement of several likely behavioral focus areas, compiled following immersions (see Results, Fig 4). Depending on how much was already known from immersion interviews, these drivers could be specific (such as risk perceptions), or could be focus areas on a broader level, such as coping mechanisms in response to stress, which in further studies could be more finely differentiated. The game was recently used to gain insight into the barriers and drivers of voluntary medical male circumcision in Zambia and Zimbabwe [29], but its mechanism is first described here. Crucially, the game requires fast decision-making, giving participants little time to deliberate, and it does not directly ask participants for their beliefs and motivations, but infers them from the choices participants make, thereby reducing social desirability bias (the tendency to select responses that show the participant in a good light [30]).

#### Decision-making game analysis

As the decision-making game asked participants to select from a set of response options, quantitative scores were obtained. However, due to the sample size, the main purpose was not quantitative inference; rather, scenarios served as discussion starters for extensive interviews after the game. Audio recordings of interviews were first transcribed and then translated to English. Qualitative transcripts were again analyzed using thematic analysis, with a coding team and process as for immersion interviews. First, the coding scheme for interview transcripts was refined from immersion results and comprised tags for each of the behavioral focus areas (Fig 4) as well as the key RMNCH behaviors, such as activities related to family planning, baby survival, breastfeeding and healthy baby. For each tag category, the insights were then summarized from the consolidated list of relevant quotes from all transcripts. Insight summaries were triangulated with the quantitative game response data for sense-making.

In a next step, to add clarity, outputs from both immersion interviews and the decision-making game were further qualitatively summarized into three main types of behavioral drivers, shown to be predictive in several models of behavior including the Health Belief Model [31, 32], Integrative Model of Behavioral Prediction [25, 33], Social Cognitive Theory [34, 35] and the Health Action Process Approach [36]. First, people are influenced by their emotional arousal state (such as stress). Second, beliefs about the threat inherent in a behavior can drive or inhibit action (risk perception). The motivation to act is also influenced by the belief that the individual will be able to affect the outcome with the given skills, barriers, and facilitators (self-efficacy).

In a final step, results from both immersion and decision-making game analysis were combined into narrative summaries (see Results), supported by quotes from immersions and post-game interviews where possible. Quotes are by staff nurses, unless otherwise indicated.

## Results

### Immersion interviews indicate overall shortfall in target behaviors

The immersion interviews allowed us to qualitatively compare the list of behaviors indicating optimal quality of care with the realities in facilities, and get a sense of the likely importance of different types of drivers and barriers to high-quality care. For instance, we found that family-planning counseling was severely lacking, or was ad hoc and arbitrary. As likely barriers, interviews indicated that religious and social norms, as well as incorrect health risk perceptions (such as the belief that IUCDs are harmful) influenced family-planning advice. As another example, staff nurses indicated a tendency not to weigh new-born babies after birth, likely driven by low risk perceptions (as many babies are underweight), a low arousal state after the delivery had been successful, low ownership and role clarity, and the social norm that small babies are not a problem. In sum, we found 17 factors likely involved in critical behaviors related to key outcomes. From these, we selected 12 factors that emerged most consistently across respondents to be tested in the decision-making game scenarios (Fig 4), encompassing a variety of external or internal areas of focus (refined from the broad structure of Fig 1):

This enabled us to then map the full set of elements described in Fig 2, from target outcomes to behavioral focus areas. Fig 5 shows the full list of behaviors of interest identified, and S1 Fig contains the complete Providers Outcomes Pathway:

### Context drives nurse behavior

As the UPP framework (Fig 1) distinguished between external context and internal evaluation of that context, we first focused on the context staff nurses operate in, and the perceptions, goals, and coping mechanisms in response to overall context (Fig 6).

Nurses faced a stressful workload and often lacked resources, support, and the respect of their supervisors. Immersion observations showed that they were also often confronted with resistance from patients, as well as interference from their families, who did not view nurses as figures of authority in the facility: “*When it comes to staff nurses*, *they [families] feel that we do not know anything*. *If they get assurance of a doctor*, *then they feel more satisfied*”. The nurses’ supervisors (LMOs or medical officers in-charge) constantly scrutinized them for adverse outcomes, which at times resulted in a culture of punitive supervision: “*We have to take the decision by ourselves and if anything goes wrong then we are answerable*. *Our seniors will ask us why we kept the case when we had an option of referring it*”. Nurses are responsible for most tasks pertaining to the delivery process at the facility. However, the outcomes of these tasks are influenced by a number of factors and people outside the nurse’s control, thereby lowering her control over outcomes. Observations and decision-making games showed that nurses perceived an overall environment of stress, a lack of control, and risk to themselves, rather than just their patients.

Corresponding to that environment, three overall themes emerge as motivators for the nurses’ behavior. First, they aim to minimize stress levels whenever possible, as high stress levels reduce the ability to cope with demanding tasks. Second, they have an overall interest in minimizing, if not completely avoiding, the attribution of blame for negative maternal or neonatal outcomes at the facility. Third, nurses seek respect and authority from patients and support staff, and seek more control over tasks for which they are solely responsible.

To meet these objectives, nurses resorted to a set of coping behaviors. High stress levels led them to fall back on simple decision heuristics and routines to manage cases. For instance, similarly to a recent quantitative study [9], we observed nurses following only one or two rather than the full set of clinical steps for some examinations, or using non-clinical criteria such as non-compliant families to make referral decisions. In the decision-making game, 13 out of 36 nurses chose workload and lack of support as a key cause of stress, while 17 chose patient family interference and resistance. Nurses sought to minimize the attribution of blame by avoiding responsibility: “*We call the LMO or doctor in case of complication*. *We play it safe; if anything happens then the doctors should not blame us*.” 13 out of 36 nurses chose workload and lack of support as a key cause of stress, while 17 chose patient family interference and resistance. Lastly, they tried to assert control and authority where possible. For example, more authority and thus control was felt overall in the labor room, which is seen as the nurses’ domain. More experienced nurses also experienced greater authority and control. Asserting control could manifest as reduced empathy with patients: “*If we are under pressure then we may talk rudely to them*”.

### Behaviors and internal drivers along the RMNCH pathway

We then examined each of the key milestones on the RMNCH pathway in detail by assessing the internal beliefs, emotions and motivations experienced by the nurses, and comparing actual with target behaviors. Overall, we found a significant gap between the theoretical roles and actual behaviors of staff nurses, which varied by behavior.

#### IFA

Counseling women on iron and folic acid (IFA) use by pregnant women is not an explicit task of the staff nurses. Nurses only (sometimes) measure the woman’s hemoglobin level at the time of delivery; antenatal care is the purview of ANMs and ASHAs (accredited social health activists, another kind of front-line worker). Nurses could use this measurement to discuss IFA use for future pregnancies, and know the importance of IFA: “*If they had done ANC properly they would have not experienced anything like this [complications that cannot be managed]”*. However, there is no risk to nurses if they do not discuss IFA, and so they rarely do.

#### Safe delivery

This area comprises the screening for and management of complications on arrival, labor monitoring and complication management, active management of the third stage of labor (AMTSL), and immediate postpartum care for the mother and the newborn including persuading women to stay for 48 hours post-delivery. There are 23 steps a nurse must complete and record in a Maternal and Newborn Case-Sheet before making the decision to keep or refer a woman. This is a high-pressure task for the nurse: “*We have to take the entire decision and if anything goes wrong then we are answerable”*, and she receives little support: “*There is an LMO in my facility*, *but she sits only in her room*, *she does not come in labor room*.*”* To cope, observations showed that nurses use heuristics, focusing on those assessments they think are critical, while ignoring the rest. In addition, they face a trade-off between the stress of convincing households to comply with the referral (“*They are not willing to go that quickly*, *they want to get the work done at CHC and they don’t go ahead”)*, and the stress of complications arising during delivery following high-risk pregnancies. Therefore, they tend to go with the less stressful option on a case-by-case basis. Referral behavior varies with risk perceptions for different conditions. In the decision-making game, 31 out of 46 nurses chose low risk perception as the cause for under-referral; on the other hand, 7 out of 10 chose the lack of support from LMOs as the cause of over-referral.

In complication management, nurses must keep track of numerous measurements and supplies. If the risk is not perceived as high, nurses try to manage complications and take pride in managing complicated cases: “*They feel that [for solving high-risk cases] they will get much appreciation and will make their own area there*. *Patients will praise them everywhere*.*”* After all, complication management is a core part of nurses’ responsibilities. In the decision-making game, 7 out of 10 chose managing complicated deliveries as a way of building reputation. However, delivery outcomes are directly and punitively attributable to her, with no support from LMOs. High level of stress increases her perception of the probability of adverse outcomes (“*Yes it affects our performance because of the work pressure and because we are under so much tension*”), leading to perceptions of a high risk to herself. Stress, for example from family backlash, can also influence self-efficacy adversely. Coping mechanisms may include seeking assistance from LMOs (23 out of 46 chose this option in the decision-making game) and choosing referral to a district hospital (19 out of 46). With high risk-perception and the resulting nervousness and low self-efficacy, immediate referral and avoidance of responsibility is common: “*They were scared of using magnesium sulphate because [its] toxicity is fatal*, *so they are very much scared*, *they will not give it”* [quote by nurse mentor].

Although it is not their direct remit, Dais (traditional mid-wives) often assist with or even conduct various aspects of AMTSL: “*she [mid-wife] learns it seeing it daily and she is old so she has experience also”*. Nurses have a low risk-perception at this stage if the delivery and the baby are normal. Post-delivery, they enter a “cognitive cold state”, and risk perceptions shift to the newborn: “*If crowning is done […] then the delivery is done*”… “*Staff feel delivery is done and their work is over*”. 16 out of 36 nurses chose low risk perception as a cause for this “cold state”; in comparison, only 9 chose mere fatigue after the procedure. This could explain why guidelines are often not followed [9]. Even though outcomes are still attributable to the nurse, Dais may assist in cutting the umbilical cord:

*At the time [of post-partum hemorrhage] the mother can go into shock*, *but the staff nurse does not bother*, *once the baby is out she hands over the cord cutting part to the Dai… [The] staff nurse feels that her job is to get the child delivered*, *so they are money-oriented*. *It is heard that they say the cord will be cut by Dai only”* [quotes by nurse mentor].

In their “cognitive cold state”, the staff nurses do not actively promote a 24- to 48-hour stay post-delivery, as they are expected to do: “*We have to keep the case for 48 hours but if they are not staying then it is their problem*”. They do not expect this guideline to be followed (“*Nobody stays back for more than 12–14 hours in the hospital*”), and other healthcare staff, such as ASHAs, may not help: *“[The] ASHA only rushes*, *and clients do not say anything*. *She has in mind that other deliveries are waiting for her*, *she has to go to other houses for check-ups*.*"* Nurses have to authorize discharge from the facility, and the probability of adverse outcomes is perceived as low:

*After 2 hours of delivery [the mother is safe)*. *In intervals of 15 minutes we keep asking her whether there is bleeding or not, or if she is facing any problem or feeling scared. For 2 hours we ask this, and if she says she is fine, that means she is safe*

Nurses experience low self-efficacy due to high resistance from households, for whom it is the norm to go home soon after delivery, and because the ASHA also leaves the facility. Additionally, the nurses consider that once the woman and baby are out of the facility, the nurse can no longer be held accountable for any negative outcomes.

#### Baby survival

Three main conditions can threaten a newborn’s survival after delivery: asphyxia, a low birth weight, and sepsis. As a positive behavior, Kangaroo Mother Care (skin-to-skin contact) aids against hypothermia and other conditions.

In asphyxia management, nurses can forget to follow guidelines due to high stress, very high risk and high urgency:

*We have been trained for various things, but these are not on our mind; we have to take care of the baby; we don’t think of our hands and safety [reasons for not following protocol on hand-washing]*.

Asphyxia management is a core responsibility of the nurse, and outcomes are directly and punitively attributable to her: *“If the staff nurses make some mistake*, *they are threatened that they will be fired*, *so they are not relaxed and able to do their work nicely”* [quote by LMO]”.

Nurses often only estimate the baby’s birth weight visually. Although it is a part of the nurses’ responsibilities to manage or refer low-birth-weight babies, birth weight is not seen as a crucial sign of the baby’s health, and the nurses’ risk perceptions are low:

*If [it has been a] normal delivery*, *then the baby is out of risk*. *Several babies are a little underweight*, *so it's ok*. *… Even a 2-kilo baby is normal*. *Sometimes there is some complication*, *otherwise they cry nicely*. *There was a 1*.*5-kilo baby*, *and that baby was crying and so active that I never felt the baby was 1*.*5 kg*.

25 out of 36 nurses thought that the norm of most babies being underweight was the main driver of not weighing the baby.

In kangaroo mother care (KMC), similar issues of low risk-perception, low ownership, and the “cognitive cold state” can prevent guidelines from being followed. KMC is also not perceived as a “hard” or “serious” medical intervention, like giving a medication: “*Even keeping [the baby] away from the mother after feeding is OK*, *not much of a wrong thing*”.

In sepsis management, guidelines for hand hygiene, clean cord care, and keeping a hygienic birth place are also often not followed due to low risk-perception and low ownership: *“[The ASHA] can tell them clean cloth should be used for the child*”. While it is part of her responsibility, the accountability of the nurse is very low, as there can be many sources of infection: “*At times it happens that the baby is alright and due to the carelessness of the family members [sepsis] occurs”*. The perception of risk to the baby’s health due to infection is high:

*If the baby is wrapped in dirty cloth*, *the cord which was recently cut would touch it*, *and the baby would get an infection … The biggest danger for new-born babies is infection*. *[Therefore] oil is not supposed to be applied*.

However, the perception of attribution of blame to the nurse herself is low due to the delayed consequences. As with KMC, nurses soon lose control over the baby, and families want to carry out their own traditional post-birth behaviors relating to cord care.

#### Breastfeeding

Nurses are expected to help initiate breastfeeding immediately after delivery, and to counsel women about exclusive breastfeeding. Nurses do insist on no external feed in the facility, and risk perceptions around external feed are high:

*It is told to [families] that they cannot give anything from outside to the baby. If [the mother] is putting oil on the placenta that is external … but if the contaminated thing is going inside [the baby’s body], that is the most dangerous thing and too much harm to the child*.

However, they do not concern themselves with trying to influence the behavior of the family at home, where early breastfeeding may not be common: “*One aunt told [me] that in her time they were not breastfeeding for 3 days*.*”* While helping to initiate breastfeeding is part of her responsibility, the nurses’ accountability and risk perception is very low: “*When delivery is done normally*, *that is the most relaxed time for me”*.

#### Family planning

The nurse’s main task in family planning is counseling and convincing women to opt for PPIUCD insertion after delivery. Overall, nurses were found to be mostly indifferent about family planning activities. One reason is the ambiguity of responsibility, which 4 out of 10 nurses chose as the reason for inadequate counseling in the decision-making game. Family planning is the role of front-line health workers (ASHAs): “*They may not listen to us [for counseling] but they will listen to ASHAs because they stay in the community”* [quote by nurse mentor]. Family planning is considered non-urgent and non-critical, and is seen as the decision of the woman and her family. Therefore, the nurses’ self-efficacy in being able to persuade women is low (5 out of 10 chose it as the reason for inadequate counseling). Despite nurses being indifferent to family planning, there can be pressure from supervisors to meet PPIUCD targets, which leads nurses to selectively target families with a high number of children, especially boys:

*We ask that if you have both boy and girl or not. Some ladies say that they only have girls as of now. In that case we cannot put pressure on them and they won’t listen to us*.

#### Healthy baby

Newborn care at home is not seen as relevant to the nurse at all. Nurses are expected to counsel women on delayed bathing, clean cord care, thermal care and hygiene, but no action is taken or effort made towards counseling on or changing household behaviors at home (“*We said that do what you want at home*, *but not at the hospital*”). Instead, decisions and behaviors regarding childcare are the family’s and the ASHA’s prerogative. There is no risk perception on the part of the nurse, as she cannot be held accountable, and she has no control over the family’s behavior at home.

## Discussion

In this study, we used novel behavioral frameworks and a combination of qualitative methods to systematically identify key contextual and internal barriers that prevent staff nurses in public facilities in Uttar Pradesh from providing optimal RMNCH-related quality of care, which is one of the most pressing problems in healthcare delivery.

### Frameworks to structure research on behaviors and associated drivers and barriers

The conceptual Framework for Unpacking Provider Practices (UPP) structuring broad *types of drivers and barriers* to provider behavior, compiled from existing literature, guided the first phase of the study (Fig 1). The UPP framework helped focus data collection and initial qualitative analysis, in two ways. First, it provided a guideline for contextual and perceptual types of drivers and barriers to probe. Second, the UPP framework helped focus initial research, as it clarified that some factors, such as structural context, as well as staff knowledge and skill, were relatively well-researched. In contrast, other factors including channels of influencers, staff intentions, beliefs, and social norms had been under-studied. We propose that the UPP framework can serve future research on healthcare providers as a template for the aspects that need to be captured to understand behavior holistically. The framework also highlights that a methods mix is needed to capture the full set of drivers. In this study, immersions and a novel decision-making game were used to capture beliefs, norms, influencer pathways, and nurse perceptions of context.

We then developed the Providers Outcomes Pathway (POP) to help identify the relevant *facility-based behaviors* to focus on, and, following initial qualitative immersion results, *refine the specific drivers* (for example, specific beliefs) that might be linked to these behaviors (Fig 5 and S1 Fig). These were then investigated in depth in the subsequent decision-making game scenarios. By narrowing down the focus of the decision-making game, informed by initial qualitative research, the POP approach aims to increase the likelihood that the drivers and barriers investigated in depth ultimately relate to target health outcomes.

### Contextualizing results

The findings of this study are applicable beyond RMNCH and have implications for primary health care systems overall. Our results highlight that an environment of stress, punitive supervision, and lack of control influences internal motivations and attitudes, and in turn behaviors, of the staff nurses. This study is the first of its kind to systematize measuring drivers of healthcare provider behavior in a comprehensive, if qualitative, way, and thus closes a key evidence gap in understanding staff behavior in public facilities. Crucially, the factors studied go beyond the resource and management-related barriers that have predominantly been the target of research [10–12]. We argue that understanding behavior on a deeper level–both contextual, and internal to the healthcare provider–will enable much more efficient development of levers for behavior change, which can then be implemented, tested, and finally scaled up. Such research could, for example, shed light on why providing protocol checklists to facility staff only had a temporary effect on adherence to these protocols [15]: the staff drivers of behavior may not be aligned with the program imperative to follow a checklist, even if it provides evidence-based recommendations.

Overall, we found that staff nurses in Uttar Pradesh have their own perspective on their jobs, distinct from program perspectives (Fig 7).

Nurses keep or refer patients using stress- and risk-driven heuristics, and while programs may prescribe guidelines for all clinical areas in the same way, nurses follow them to a different degree depending on whether the behavior is “hot state” (anything to do with the delivery itself, which is her core responsibility) or “cold state” (behaviors after the delivery is over). For example, while programs expect nurses to monitor and counsel post-delivery, those behaviors are given short shrift by nurses who do not see them as their primary responsibility.

### Recommendations: Designing effective behavior levers

Our findings show that the resources that facility-based nurses in Uttar Pradesh could offer are not tapped in an optimal way. Interventions to improve quality of care should initially focus on four levels, three of which focus on the context surrounding nurses (Fig 1). First, across facilities, structural gaps should be closed as a prerequisite for an environment in which nurses can perform their duties well. This includes a reliable supply of commodities, adequate staffing levels, system and process management tools, transport to and between facilities for families, and resources available to families to stay after delivery. In Uttar Pradesh, there have been significant structural improvements over the last years via government and donor investment. These improvements are necessary and need to continue, but are not sufficient to optimize quality of care. Second, systemic change poses both the greatest difficulty and the largest opportunity for change. Staff nurses work in an environment of low accountability and support; where supervision occurs, it can be punitive. Any effective change to such large-scale processes, but also cultural shifts regarding accountability and blame, can only be implemented at the policy level external to individual facilities. However, today the majority of investments focus on interventions within facilities. It would be easy to provide a list of recommendations for further training or education of staff nurses, or create tools such as improved case-sheets or “muscle memory” drills for asphyxia management. However, we find that the culture of stress, blame, and lack of control is pervasive, and while improving the knowledge and skills of nurses has improved quality of care in many clinical areas [9], key gaps remain. Change therefore needs to start beyond the nurse’s personal level to be effective, and we argue that otherwise, any return on interventions at the nurse’s level alone would be marginal. Measures should be identified to hold medical officers in-charge accountable for managing quality of care, resourcing to facilitate family stay after delivery, and overseeing clinical decision-making. They should experience reward for compliance and consequences for non-compliance. Protocols for team interaction between the different roles, as well as regular case review meetings, would further strengthen support and accountability. In the labor room, protocols for respectful maternal care, and a birth companion as an individual helper for the patient, would provide further accountability for the nurse as well as support for the woman. The birth companion could be involved from the antenatal care stage for maximum awareness of the woman’s medical situation. As nurses are in a post-delivery “cognitive cold state”, an additional maternity ward role could be created to counsel and monitor mothers and babies after delivery, as well as build a bridge between communities and facilities via front-line workers. Existing RMNCH counselors could also be activated to fill that role, for example on family planning advice, and offer group and individual counseling for families. Consequently, the staff nurse’s role could be focused, and her efforts concentrated on critical delivery-related behaviors.

Third, community norms and expectations must be shifted to align with the behaviors expected of the staff nurse. As we found significant family interference and backlash in several areas, families should be prepared during antenatal care check-ups for facility delivery, the possibility of referrals, and post-natal care behaviors. This preparation is especially important as some beliefs around behaviors are strongly ingrained, and the stressful time around delivery makes it difficult for families to focus on anything beyond immediate and familiar steps. For example, community health workers should prime families on contingency planning, such as where they might need to go in case of complications and what resources they might need to stay in the facility after delivery. They should also encourage families to follow the clinical decisions of nurses and other providers.

Fourth, within the facility the key internal drivers of the nurse should be addressed. For example, to mitigate stress in emergency situations, simulated emergency drilling practices that practice “muscle memory” should be held regularly. In order to maximize nurses’ self-efficacy, case sheets should be improved to become user-friendly job and memory aides, instead of being filled in after the situation has passed. It should focus on including only critical measurements useful for nurses while they are managing cases, distinguish sections that help the nurse do her job from those that are for record keeping, and build shared ownership for clinical decision-making by including supervisor sign-offs. Risk communication should be improved across the board by leveraging evidence-based research, so the patient-centered risks around hygiene, anemia, referral practices, clinical assessments, delivery and post-delivery behaviors become more salient to the nurse. Clear causal links between each step of medical interventions and outcomes need to be established using real-life examples, emergency drills, and case narratives. Communicating risks should always be tightly linked to the actions the nurse can take to mitigate these risks. Health risks resulting from not following guidelines can also be emphasized as risks to the nurse herself: for example, ignoring anemia increases the likelihood of labor room complications, the stress of which is already keenly felt by nurses and which they are motivated to avoid.

### Limitations and next steps

While this study outlines a rich landscape of barriers to and drivers of behavior, it has several limitations. First, a relatively small sample size in a single clinical area could limit generalizability to other fields, and is not sufficient to explain the substantial heterogeneity between the quality of care in different facilities. Second, as this study is exploratory and qualitative, it is currently unknown how different influences on behavior interact, and which factors are predominantly important in influencing behavior. This will be addressed in upcoming quantitative research, which will quantify both external and internal influences on behavior for the first time and model linkages and interactions between those drivers. For example, it is currently unknown whether and to what degree training or skill-building can overcome low risk-perceptions or self-efficacy. Future studies should also investigate the entire system network and all types of providers within it, not just staff nurses. Furthermore, it also remains to be seen which types of interventions will succeed in changing suboptimal practices, and to what extent. Careful development and rigorous evaluation of interventions is therefore indispensable, while keeping in mind that blanket targeting of facilities and providers may not be the optimal solution. Instead, further research is required to identify those facilities that are more likely to achieve target health outcomes in response to interventions, and to segment healthcare providers into sub-groups that would benefit from tailored support. At this point, our research does not account for sub-groups of providers, for example staff nurses with exceptionally low or high risk-perceptions or self-efficacy. Segmentation on such behavioral drivers, and by facility readiness, is likely to enable much more effective intervention design [29].

As well as potential interventions and frameworks to guide the research design process, this study also offers a thorough application of a novel method, a decision-making game, to study behavior in a field setting with lay participants. While based on general principles of behavior, this method can be customized to any investigation, as we have recently shown in a very different application around voluntary medical male circumcision [29]. The scenario-based decision-making game could also be scaled to a larger sample and develop from a qualitative sense-making into a quantitative hypothesis-testing approach. As the literature on understanding provider behavior shows, this field is in dire need of tools that go beyond direct surveys of personal opinion and experience, which are notoriously prone to self-report biases [30]. The tools mentioned here will enable researchers to systematize their understanding of behavior, and provide useful input into understanding sub-optimal quality of care.

## Supporting information

S1 Fig Providers Outcomes Pathway (POP). Full pathway from key causes of maternal and neonatal mortality, to preventive and curative behaviors (within facilities), to field data on those behaviors, and finally focus areas forming the basis of the decision-making game.
(PDF)

S1 Table Block and facility selection for immersions. (XLSX)

S2 Table Data collection and coding team characteristics. (XLSX)

S1 File Immersion discussion guide Hindi and English, staff nurse template. This template was adapted ad-hoc for other facility-based roles.
(PDF)

S2 File Facility selection (TSU) for decision-making game. (XLSX)

S3 File Facility selection (Non-TSU) for decision-making game. (XLSX)

S4 File TSU CHC facility parameters for decision-making game. (DOCX)

S5 File Non-TSU CHC facility parameters for decision-making game. (DOCX)

S6 File TSU DH PHC facility parameters for decision-making game. (DOCX)

S3 Table Staff nurse selection per facility type for decision-making game. (XLSX)

S7 File Full decision-making game scenarios. (XLSX)

S8 File Decision-making game discussion guide, Hindi and English. (DOCX)

S9 File COREQ (COnsolidated criteria for REporting Qualitative research) checklist. (PDF)
