# Supplementary figures and images for: Categorizing and assessing comprehensive drivers of provider behavior for optimizing quality of health care
Source: PLoS One. 2019 Apr 17;14(4):e0214922. doi: 10.1371/journal.pone.0214922 (PMC6469845; doi:10.1371/journal.pone.0214922)

FACILITY LANDSCAPE

NEONATAL MORTALITY

MATERNAL MORTALITY

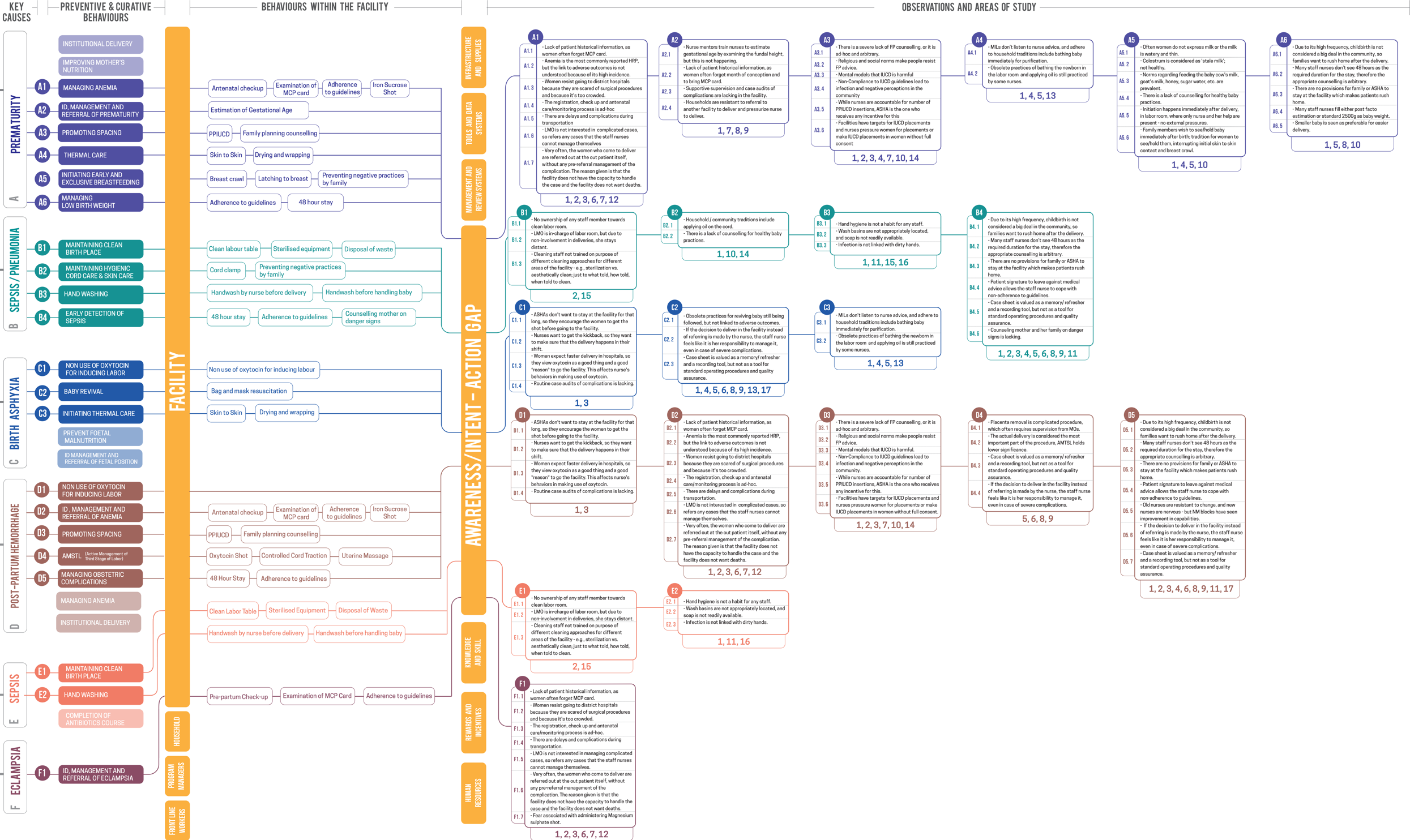

Supplement: S1 Fig — Full pathway from key causes of maternal and neonatal mortality, to preventive and curative behaviors (within facilities), to field data on those behaviors, and finally focus areas forming the basis of the decision-making game. (PDF) [file pone.0214922.s001.pdf]
