# Supplementary material for: Categorizing and assessing comprehensive drivers of provider behavior for optimizing quality of health care
Source: PLoS One. 2019 Apr 17;14(4):e0214922. doi: 10.1371/journal.pone.0214922 (PMC6469845; doi:10.1371/journal.pone.0214922)
Supplement: S1 File — This template was adapted ad-hoc for other facility-based roles. (PDF) [file pone.0214922.s004.pdf]

Staff Nurse:

Start by making a casual conversation with the interviewee. Ask questions about their background, family, age and occupation.

Try to ask questions that will lead to a story, not merely an answer.

Do not interrupt the interviewee when he/she is answering. Let them finish their answer.

Make sure the transitions between the questions are seamless.

Empathise with the interviewees.

The following topics have to be covered in the interview.The detail questions under each topic have been attached in the appendix.

Embrace the silences. Even if they are awkward.

For every response, ask for examples or to narrate experiences.

Primary Discussion Guide

General questions: आप कब से नर्स हैं?

- How long have you been working as a nurse?
- Which course did you undertake for becoming a nurse?
- नर्स बन्ने के लिए आपने क्या पढाई की?
- How long have you been working at the current facility?
- आप इस अस्पताल में कब से हैं?
- Who do you have in your family?
- आपके घर में कौन कौन है?
- What motivated you to become a nurse?
- आपने यह काम क्यों चुना?

Job Profile and Work Routine: अपने दिन के बारे में हमें बताइए

- What does an average day look like? Which activity takes most of your time in the day
- आपके किन कामों में सबसे ज़्यादा समय लग जाता है?
- Apart from routine work what are the occasional (monthly, quarterly) responsibilities you have to undertake?
- रोज़ के कामों के अलावा आप किन चीज़ों के लिए ज़िम्मेदार हैं?
- Which aspects of your job are you most satisfied with?
- Which aspects of the job are you least satisfied with?
- Do you have appropriate equipments, etc. for your job? What would you like to add?
- काम के लिए आपको जिन चीज़ों की ज़रुरत पड़ती है, क्या वह उपलब्ध रहती है?
- Do you think the facility is appropriately staffed? What kind of staff do you think should be added on?
- क्या अस्पताल में काम के हिसाब से पूरा स्टाफ है?

- What data collection, entry and compilation do you have to undertake? What can be improved about this?
- आपको किस तरह का पंजीकरण, रजिस्ट्रेशन और एंट्री भरनी पड़ती हैं? इन कामों को बेहतर कैसे किया जा सकता है?

Relations with Staff
- How do you interact with most during your work?
- आप अस्पताल के किन स्टाफ के साथ सबसे ज़्यादा काम / बात करती हैं?
- Who do you seek support from in a challenging case?
- मुश्किल केस आने पर आप किस्से मदद मांगती हैं?
- Is the administration responsive to your concerns? Which aspects of administration can be improved?
- आपकी चिंताओं और तकलीफों को लेके अस्पताल का मैनेजमेंट क्या करता है?
- How often do you interact with MOIC? How are your relations?
- MOIC आपके काम में किस तरह का किरदार निभाते हैं?
- How often do you interact with frontline workers? How are your relations?
- आपके आशा, ANM और आंगनवाड़ी महिलाओं के साथ किस तरह का ताल्लुकात रहता है?

Training and Casesheets
- How often do you interact with nurse mentor? How are your relations?
- आपकी नर्स मेंटर से किस तरह की बात चीत रहती है?
- How often do you receive training from nurse mentor?
- नर्स मेंटर आपको कितनी बार और कब कब ट्रैन करती हैं?
- Which aspects of training have you benefited most from?
- ट्रेनिंग से आपने तीन सबसे महत्वपूर्ण चीज़ें क्या सीखी हैं?
- Which aspects of the training can be improved?
- ऐसी तीन सुझाव दीजिये जिससे ट्रेनिंग बेहतर हो सकती है
- Do you use casesheet? How long have you been using it?
- What kind of problems did you face in the initial adoption of casesheet?
- शुरू में केस शीट को इस्तमाल करने में क्या मुश्किल लगता था?
- What are the three most useful aspects of the casesheet?
- केस शीट किन तीन कामों को आसान बनाती है?
- What are the three aspects of the cassette which can be improved?
- केस शीट में तीन कौनसे सुधार लाये जा सकते हैं?

Delivery and Newborn Care
- (AMTSL) Describe the entire procedure of the journey from when the woman comes in for delivery till she leaves.
- किसी एक डिलीवरी केस का उद्धरण लेके, शुरू से लेकर अंत तक हमें उसकी प्रक्रिया समझाइये
- Describe the management, care and referral of High Risk Pregnancy
- उच्च खतरे वाली गर्भावस्था की पूरी देखभाल के बारे में बताइए
- How long do the women usually stay for after delivery? What % of women stay for 48 hours?
- डिलीवरी के बाद महिलाएं आम तौर पर, कितनी देर तक अस्पताल में रूकती हैं? कितनी % पुअर 48 घंटे रूकती हैं?
- How do you deal with each of these complications:

- बच्चे को अगर अस्पिक्सिा हो तो, आप क्या करती हैं?
- Asphyxia
- Low Birth Weight -- वज़न कम हो तो?
- Infection Management -- बच्चे को इन्फेक्शन से बचाने के लिए?
- Describe what you do in terms of counselling and delivery of family planning services.
- परिवार नियोजन के लिए आपका क्या किरदार रहता है?
- Walk us through your thermal care procedure. Do families accept this procedure? If not, in what ways do they treat the child in this condition?

Families and Counselling

- What do you counsel the woman/family about? Which stage do you counsel at?
- आप महिलाओं और उनके घरवालों को किस तरह की सलाह देती हैं?
- What kind of activities and messages do you receive pushback from families from?
- In an average household of newborn, who is most receptive and least receptive to your advice?
- नवजात बच्चे के घर में कौन आपकी सलाह सुनने के लिए सबसे ज़्यादा तैयार रहता है? और सबसे काम तैयार?
- Which traditional beliefs and norms interfere with medical procedures and behaviour change?
- ऐसे कोई रिवाज़ या धारणाएं हैं, जो मेडिकल सलाह में बाधा डालती हैं?
- Which techniques do you use to deal with pushback?
