# Supplementary material for: Categorizing and assessing comprehensive drivers of provider behavior for optimizing quality of health care
Source: PLoS One. 2019 Apr 17;14(4):e0214922. doi: 10.1371/journal.pone.0214922 (PMC6469845; doi:10.1371/journal.pone.0214922)
Supplement: S4 File — (DOCX) [file pone.0214922.s007.docx]

**The different performance parameters considered to classify performance of TSU facilities are:**

1. Complication management of maternal complications^[[1]](#footnote-1)^
2. Complication management of newborn complications^[[2]](#footnote-2)^
3. Availability of critical resources (Oxytocin, Misoprostol, Anti-hypertensive, Magnesium Sulphate, IV fluids, Partograph, Antibiotics-5X5 matrix) essential to manage maternal complications
4. Availability of critical resources (Vitamin K, Mucus extractor, Bag & Mask, Clean linen/towels, Sterile cord cutting equipment, Newborn care corner, Radiant warmer) essential to manage newborn complications
5. SN/ANM trained in SBA (Skilled Birth Attendance Training)
6. SN/ANM trained in NSSK (NavjaatShishu Suraksha Karyakram, newborn care and resuscitation)

**The corresponding indicators of the aforementioned parameters used are:**

1. % maternal complications **managed at facility and sent home healthy** and **managed at facility, referred to another facility and sent home healthy**
2. % newborn complications **managed at facility and sent home healthy** and **managed at facility, referred to another facility and sent home healthy**
3. % availability of critical resources to manage maternal complications
4. % availability of critical resources to manage maternal complications
5. % SN/ANM trained in SBA
6. % SN/ANM trained in NSSK

**Population size**

151 CHCs from TSU blocks. (15 CHCs had to be dropped owing to missing data)

**Groups to be selected:**

1. One group of 8 “good” performing TSU CHCs
2. One group of 9 “average” performing TSU CHCs
3. One group of 8 “poor” performing TSU CHCs

**Methodology parameters**

Note: Not all of the parameters listed below have been used while selecting each group. The parameters used for each group have been specified in the next section.

1. Classification of facilities based on performance indicators

*Step 1: Classification of facilities indicator wise*

Note: Different indicators have different cut off points. The distribution of each indicator has governed the cut off points.

- 1. Good if indicator =100, Average if indicator between 71 and 99 and poor if indicator <70
  2. Good if indicator =100, Average if indicator between 71 and 99 and poor if indicator <70
  3. Good if indicator >85, Average if indicator between 65 and 85 and poor if indicator <= 65
  4. Good if indicator >85, Average if indicator between 65 and 85 and poor if indicator <= 65
  5. Good if indicator >50, Average if indicator between 10 and 50 and poor if indicator <= 10
  6. Good if indicator >50, Average if indicator between 10 and 50 and poor if indicator <= 10

*Step 2: Overall classification of facilities:*

Good: if 4 or more of the 6 indicators are “good”

Poor: if 3 or more of the 6 indicators are “poor”

Average: all other remaining combinations

1. Geographical consideration for logistics: facilities selected in one group to be as close to each other as possible
2. Ranking of facility based on aggregate score of 6 indicators (all given equal weight)
3. Average monthly admission load: High load CHC if average monthly admission load > 200; Medium load CHC if average monthly admission load is between 50 and 200; low load CHC if average monthly admission load <=50

1. Maternal complications include PPH, Eclampsia, Maternal Sepsis and Obstructed labour [↑](#footnote-ref-1)
2. Newborn complications include Preterm/Low birth weight, Asphyxia and Newborn Sepsis [↑](#footnote-ref-2)
