# Supplementary material for: Categorizing and assessing comprehensive drivers of provider behavior for optimizing quality of health care
Source: PLoS One. 2019 Apr 17;14(4):e0214922. doi: 10.1371/journal.pone.0214922 (PMC6469845; doi:10.1371/journal.pone.0214922)
Supplement: S5 File — (DOCX) [file pone.0214922.s008.docx]

# Selection Procedure for Ethnolab Groups: Non TSU CHCs

**Groups to be selected:**

1. One group of 8 “good” performing Non-TSU CHCs
2. One group of 7 “poor” performing Non-TSU CHCs

**The performance parameters considered to classify performance of Non-TSU CHCs are:**

Composite score of

- 1. Normalised scores of Staff Nurses’ skills
  2. Normalised scores of Staff Nurses’ practices
  3. Normalised scores of essential equipment availability
  4. Normalised scores of essential drug availability

*Note: Scores for availability of drugs, equipment, skills and practices were generated using the PCA approach*

**Population size**

20 CHCs from Non-TSU districts

*Note: No other reliable data source available for Non TSU districts’ CHCs on facility performance, hence forced to consider QoC data, which has limited data points.*

Group 1: One group of 8 “good” performing Non TSU CHCs

8 “good” performing Non TSU CHCs with the highest composite scores are selected.

Group 2: One group of 7 “poor” performing Non TSU CHCs

7 “poor” performing Non TSU CHCs with the bottom lowest composite scores are selected.
