# Supplementary material for: Categorizing and assessing comprehensive drivers of provider behavior for optimizing quality of health care
Source: PLoS One. 2019 Apr 17;14(4):e0214922. doi: 10.1371/journal.pone.0214922 (PMC6469845; doi:10.1371/journal.pone.0214922)
Supplement: S8 File — (DOCX) [file pone.0214922.s012.docx]

Ethnolab: Facility Scenarios Discussion Guides

| Hypotheses/Principles | Scenario Narrative | Discussion Guides for In-Depth Interviews |
| --- | --- | --- |
| S1: Causes of Stress  - Working conditions - long, ambiguous shifts, inadequate resources and support  - Patient and family expectations, risks and management  - Pressure of targets and accountability (externalize in framing) | Rita, Gita and Sita are staff nurses at Community Health Centres in three different blocks. All three of them are facing some problems at work. A. Rita's facility is understaffed, and hence she has to work longer shifts. Her shifts keep changing too, and she receives little or no support in her work. B. Gita often has to deal with unreasonable demands and misdemeanours of the patient's family members, who do not understand risks and procedures, and create disturbances and tension around the labour room. C. Sita's facility faces constant scrutiny from the district authorities, and the accountability for facility performance often falls on her. She frequently gets reprimanded by her seniors for facility not meeting targets. Who among the three staff nurses is most likely to be most stressed due to the problems at work? | Can you describe a time that you were stressed? What caused it, what led to it, what contributed to it? How do you tackle this? What helped you overcome the stress? Did stress affect your work and performance? What are the most important targets? Which targets are hard to achieve and why? How are these targets communicated to you? Who monitors your performance against the targets? Which behaviours and demands of patients stress and irritate you the most? How do you handle difficult patients and their families who come into the facility? How do you manage their expectations? What kind of a relationship do you maintain with patients and their families while they are at the facility? (do they keep interaction to a minimum. are they friendly with them, are they polite and have patients taken advantage of them in such cases?) Are your shifts fixed or do they keep changing? How do you manage your high workload and long shifts? Who do you receive most support from in highly stressful situations? |
| S2: Over-referral  - Authoritative monitoring and accountability  - Workload and stress  - Lack of coordination with LMO | Rita, Gita and Sita are staff nurses at Community Health Centres in three different blocks. All three of them are evaluating whether to refer women with moderate anemia to district hospital or to conduct the delivery at CHC.  A. Rita's facility faces constant scrutiny from the district authorities, and the accountability for facility performance often falls on her. She frequently gets reprimanded by her seniors for adverse outcomes.  B. Gita's facility is underresourced for the footfall it receives. So the staff has high workload and is often stressed.  C. Sita doesn't have a good communication and working relation with the Lady Medical Officer at her facility. She is reluctant to approach LMO for support in managing HRPs and complications.  Who among the three staff nurses is most likely to refer the moderate anemia case to the district hospital instead of conducting delivery? | What is the protocol for admission of a pregnant woman in the facility? Where does she go first?  Take us through a typical pre-partum check up at your facility  - What? What are the various parameters that are checked? Medical history?  - Where? Does it shift or is it a fixed place?  - Who checks the vitals of the patient? Who is in-charge?  - Who makes the decision of referring or not referring the patient in case there is a complication?  - How is the decision made? What is the criteria used to make the decision?  - Before referring the patient out, what are the arrangements that have to be made?  - Where and how are the vitals recorded? By whom? When and how do you use these records during delivery?  - What kind of problems do you face in conducting pre-partum checkups and referring HRPs? What do you do to tackle these problems? Give examples.  Have there been cases when the referred patient has faced severe complications before reaching the DH? What do you think caused the complication? How could it have been avoided? Would you have done anything differently?  Is there a high workload at your facility? How does the workload impact the referrals and related decision making?  What kind of coordination is required with LMO in the referral process and decision? Is there a fixed protocol? Who else is involved/assists in this process? Have you faced any problem in coordination? What do you do to tackle these problems? What do you do in the absence of LMO/assistance? Give an example of a case wherein you approached LMO for help in referral/management? |
| S3: Under-referral  - Reputation for management of high risk and complicated pregnancies  - Low risk perception  - Reluctance of patients | Rita, Gita and Sita are staff nurses at Community Health Centres in three different blocks. All three of them are evaluating whether to refer women with eclampsia to district hospital or to conduct the delivery at CHC.  A. Rita feels that conducting complicated/risky deliveries will earn her respect and good reputation among community and ASHAs.  B. Gita feels confident about managing eclampsia cases. She has managed many such cases in the past and delivered successfully without complications.  C. Sita feels that it is redundant to refer patients to district hospital because the households always resist going to district, and insist/demand that the delivery be conducted at CHC.  Who among the three staff nurses is most likely to conduct the delivery at CHC and not refer to District Hospital? | What is the protocol for admission of a pregnant woman in the facility? Where does she go first?  Take us through a typical pre-partum check up at your facility  - What? What are the various parameters that are checked? Medical history?  - Where? Does it shift or is it a fixed place?  - Who checks the vitals of the patient? Who is in-charge?  - Who makes the decision of referring or not referring the patient in case there is a complication?  - How is the decision made? What is the criteria used to make the decision?  - Before referring the patient out, what are the arrangements that have to be made?  - Where and how are the vitals recorded? By whom? When and how do you use these records during delivery?  - What kind of problems do you face in conducting pre-partum checkups and referring HRPs? What do you do to tackle these problems? Give examples.  How many HRP cases do you get in a week? How many are moderate and how many are high risk? Which cases do you see the most - prematurity, anaemia, eclampsia? Which type of HRPs do you refer the most? Which HRP condition can you manage comfortably?  Are patients/families reluctant to be referred out of the facility? Why? How do you manage such patients? Does this affect the referral process in any way?  How do families react to complicated deliveries? In case a complication arises, what is the family's reaction to the nurse?  What do you think is the main reason that other nurses under-refer HRP cases? |
| S4: Complication Management  - Refer to casesheet  - Seek assistance from LMO  - Refer to District Hospital | Rita, Gita and Sita are staff nurses at Community Health Centres in three different blocks. All three of them encounter cases of post partum haemorrhage while conducting deliveries, respectively. Due to heavy bloodloss, they have to quickly make the decision of how to manage the complication.  A. Rita uses the casesheet to find the correct protocol to manage such complications because she feels that it has to be managed as quickly as possible to avoid further complications.  B. Gita seeks assistance from the LMO because she feels the need for assistance and support in dealing with such a complication.  C. Sita immediately refers the case to District Hospital, as she feels that such complicated cases cannot be managed at the CHC.  Which staff nurse's action is most likely to be successful in managing the complication? | What kind of complication do you encounter during deliveries? Whose role is it to take care of a complication when it arises during delivery? How do you manage complications when they arise? (call for LMO or nurse mentor, do they try to manage on their own, what aides do they use if they try to manage on their own?)  If you can't get help from your LMO/NM, how do you manage the complication? Do you feel confident managing complications? Give us an example of a case you didn't feel confident in managing the complication yourself and how did you go about it? What kind of existing aides did you use then? What could have been done to prevent the complication?  Is casesheet helpful in managing complicated or unfamiliar cases?  How did you feel about having to take care of a complication without any support? What was your thought process when you had to do this? Among the available team members whose help did you take and how?  Give us an example of a time that the LMO helped you in a complication? How did you approach the LMO? |
| S5: Attribution of Adverse Outcomes  - Negligence/ignorance of household and ASHA  - Lack of training and assistance provided to her  - High number of deliveries, some of them will lead to adverse outcomes | Mala is a staff nurse at a Community Health Centre. During one of the deliveries recently, she was unable to save the baby. This incident disturbed her and she has had some thoughts about it repeatedly since then.  A. The behaviours and decision of the household and ASHA are responsible for such incidents. They do not realize the value of many procedures and behaviours, e.g. if ANC is done properly, such incidents can be prevented.  B. The facility is understaffed and the training provided to her is inadequate. Due to the lack of support and skills in conducting the delivery, such incidents are bound to happen.  C. She has conducted a hundreds of deliveries at the facility, but only a few have resulted in death of newborn. Compared to the number of successful deliveries, a few incidents are not too bad.  Which of the these thoughts is most likely to provide consolation and relief to Mala? | What is the biggest threat to a newborn's health within the first couple of hours of birth?  How many cases of Asphyxia do you encounter in a week? Do you feel confident in managing birth asphyxia cases? Which is th most difficult part of managing Asphyxia?  What are the main reasons that complications lead to negative outcomes? Why do you think that is? What can help prevent these?  Give us an example of a bad outcome. What do you think caused it? Usually in this case, what is the SOP for managing it, and why did not work this time? What could have been done to prevent it?  Do families cooperate with your advice -- out of 10 women how many listen to you? If not, does the ASHA help in making them listen to you? What else could make them cooperate with you? Who does the family listen to most: ASHA, you, or someone superior?  Do you feel confident managing complications? Do you think you need more training or support regarding some procedure? |
| S6: Strategies for Improving Risk Perceptions  - Making adverse outcomes more vivid  - Make outcome probabilities more vivid  - Make causal links more vivid through narratives | Rita, Gita and Sita are staff nurses at Community Health Centres in three different blocks. All three of them were recently trained as master trainers, to train other nurses on managing pre-term births. Each of them has a distinct approach to communication risks of preterm birth while training.  A. Rita talks about the extremely high number of newborn deaths linked to preterm deliveries in the state and the country. She tries to drive the point across that pre-term delivery is the biggest cause of neonatal mortality.  B. Gita tries to communicate the frequency of newborn deaths caused by preterm deliveries. She tells them that if a facility delivers 1000 babies in a year, each month there would be one death due to preterm delivery, on average.  C. Sita talks about her own experiences with complications and deaths due to preterm delivery. She narrates stories of some deaths at her facility due to preterm delivery and her failure to manage and prevent deaths.  Who among the three staff nurses is most likely to successfully communicate the risks of preterm delivery during training? | How many HRP cases do you get in a week? How many are moderate and how many are high risk? Which cases do you see the most - prematurity, anaemia, eclampsia? Which type of HRPs do you refer the most? Which HRP condition can you manage comfortably? |
| S7: Driving Caseshseet Compliance  - Easier to understand - more pictorial, less text  - Shorter, only important information  - Split into 3 smaller sheets to be filled before and after delivery, and at discharge separately | Rita, Gita and Sita are staff nurses at Community Health Centres in three different blocks. At their respective facilities, some changes have been introduced to the casesheet.  A. At Rita's facility, the casesheet was made easier to understand by reducing text and introducing pictorial/graphic representations.  B. At Gita's facility, the casesheet was made shorter by focusing on only the most important information and activities, and deleting the rest from the casesheet.  C. At Sita's facility, the casesheet was split into three smaller sheets, one to be filled before, one for after delivery, and one at discharge, respectively, so that the nurse rembers to use it at the right time.  Who among the three staff nurses is most likely to favour the changes and start using the casesheet more? | How many casesheets do you fill in a day? Do you fill casesheet during delivery, immediately after delivery or at the end of the day? Dos fillign the casesheet add to your workload?  What are the most useful parts of the casesheet? What are the least useful parts of casesheet?  Do you require any support or assistance while filling the casesheet? Do you understand the casesheet fully?  Do you always remember to fill the case sheet, or do you need to be reminded often? |
| S8: Post Delivery Mental State  - Low risk perceptions  - Cognitive fatigue from stress  - Low ownership | Rita, Gita and Sita are staff nurses at Community Health Centres in three different blocks. All three of them have just conducted a delivery successfully. Now they are considering whether they should leave the placenta removal responsibility to the Dai and move on to the next patient.  A. Rita feels that after a successful delivery, the major risk for mother and child health is over, and Active Management of Third Stage of Labour is a straightforward and riskless procedure, that can be easily managed by the Dai.  B. Gita feels quite tired immediately after the delivery, due to the complicated procedure and high risk environment, so she likes to delegate activities immediately after delivery to Dai.  C. Sita feels that the most important responsibility of the nurse is delivery, and the support staff is there to help them with other less critical activities.  Who among the three staff nurses is most likely to leave the placenta removal/AMTSL responsibility to the Dai? | During the time the woman is at the facility, what period is the most important? Stressful? What is the most relieving? Following delivery, what is the most urgent thing to take care of?  What are your responsibilities during the delivery and post delivery processes? Which responsibilities does the Dai support you in? Which responsibilities do you trust the Dai to manage by herself?  At what point of time do you think that the newborn child is safe and out of danger? What about the mother?  Walk us through what happens right after the child is born? How do you remember what you have to take care of after delivery?  Is there a check-list you refer to for post-delivery procedures? At what times do you use it?  Do you feel that there is low motivation for post-delivery procedures like thermal care, breastfeeding? What causes this?  Do you face any comlication during removal of placents? How do you deal with these? Can the Dai manage placenta removal without you? |
| S9: Reasons for Inadequate Counselling  - Ambiguity about responsibility  - Low self-efficacy  - Lack of time and appropriate context for counselling | Rita, Gita and Sita are staff nurses at Community Health Centres in three different blocks. All three of them are considering whether they should spend more time on counselling pregnant women about spacing and contraception methods, but they have certain doubts.  A. Rita feels that such family planning counselling is ASHA's responsibility, and they even receive incentives for it.  B. Gita feels that counselling is ineffective because the households have their own beliefs and norms regarding family planning, and they do not listen to doctors and nurses regarding this.  C. Sita feels that because the families are always in a rush and stressed during delivery, and too distracted and tired after it, it is not the appropriate time and place to counsel.  Who among the three staff nurses is least likely to spend more time on family planning counselling? | Give us an example of a counseling session you did, and how did it go? What worked and what didn't work? Why didn't it work? Who do you think should be doing the counseling? Why --> do they listen to them?  Which are the key topics that you counsel women about? Regarding which topics do you feel most resistance from the family?  What can you do to make sure the woman is more informed and has enough information for a good decision making process?  About how many people do you counsel in a week? How many deliveries? When do you counsel the woman? What do you think is the right time to counsel a woman?  Give us an example when you had to try very hard to convince the family and it worked? Usually what are the biggest challenges you face when trying to counsel the woman? The family? How have this changed your approach for counseling?  How receptive are the patients to family planning advice? Who is the best person in the family to counsel about FP? Do they listen to ASHA or you regarding this? Which beliefs and attitudes make people resistant to family planning? |
| S10: Perception of Value of Casesheet  - Accountability  - Remembering small things otherwise forgotten  - Managing complicati | Rita, Gita and Sita are staff nurses at a Community Health Centre. They are discussing how they use and value the casesheet.  A. Rita says that the casesheet is very useful because if you fill it every time, then any complication later in the mother or child's health cannot be blamed on you.  B. Gita says that the best use of casesheet is to remember minor but important things that you might otherwise forget during a delivery due to distractions and stress.  C. Sita says that the casesheet is most useful in complicated cases which you wouldn't have seen or managed before. In such cases, the casesheet can tell you what to do.  Who among the three staff nurses is likely to use the casesheet the most? | Do you believe the case-sheet is an important document? Why?  Why do you think the case-sheet was introduced in the facility?  Have you done anything to improve the value of the casesheet? What tricks have for you, have you shared these in your facility? How has the response been?  What happens / what are the consequences if you don't fill the case sheet?  Can you describe an example when the case sheet was not filled and nothing happened? Can you tell us about a time that the case sheet was helpful to you in managing a case?  Does the casesheet help you in recording adverse and uncooperative practices of the families? |
| S11: Perception of Value of Nurse Mentor  - Refreshing knowledge and skills  - Helping with managing complication  - Helping with communication within facility and getting things done | Rita, Gita and Sita are staff nurses at Community Health Centres in three different blocks. They are discussing how the nurse mentor helps them and improves their performance.  A. Rita says that the nurse mentor has helped her to continue to improve her knowledge and develop new skills regarding mother and child health, beyond the education and training she received.  B. Gita says that the nurse mentor has helped her in managing complicated cases and new kind of cases which she used to be unable/reluctant to manage or make mistakes while managing.  C. Sita says that the nurse mentor has helped by understanding her needs and problems, and raised these issues with appropriate authorities and staff to resolve some of them.  Who among the three staff nurses is likely to value her nurse mentor the most? | Who is a part of your team? What are their roles?  Whose support do you have most when you need it?  In what areas of work do you think teamwork can make things smoother and better?  In what areas of work do you feel unsupported, and what can be done to make that better for you?  What areas of work are already working well because of having a strong team?  Give us an example of a time that the nurse mentor proved to be useful and helpful in a case? Give us an example when the nurse mentor could have helped but they didn't. Which skills of yours have improved the most due to Nurse Mentor? In which skills did you not require much support?  In what cases do you go to the MO or LMO for help? In what cases do you go to the nurse mentor for help? |
| S12: Risk Perception Regarding Practices of Family at Facility  - Feeding something  - Applying oil on the cord  - Taking baby away from mother immediately after delivery | Rita, Gita and Sita are staff nurses at Community Health Centres in three different blocks. All three of them have just conducted a delivery successfully. But after the delivery, they observe some behaviours of the respective families which annoy them.  A. Rita sees the sister-in-law of the mother feeding something to the newborn.  B. Gita sees the grandmother of the newborn applying oil on the cord.  C. Sita sees the father of the child take the newborn away from the mother immediately after delivery.  Who among the three staff nurses is likely to be most annoyed by the respective families' behaviour? | Which are the worst behaviours of family? Which are the most commonly observed adverse behaviours?  Do you see value in any of the behaviours and beliefs? How do you think are these beliefs useful to the community?  Which are the key topics that you counsel women about? Regarding which topics do you feel most resistance from the family?  What can you do to make sure the woman is more informed and has enough information for a good decision making process?  In what ways do the patients and their family co-operate in post delivery procedures?  - Do you face pushback from families on this protocol? What for? How do you react to such situations?  - What are the main reasons for women to not follow the instructions?  - What do you think about the existing norms around thermal-care and breastfeeding? Are there any community norms which according to you are valuable and useful? |
| S13: Valued/Desired Reputation Aligned with Patient Expectations:  - Reputation for speedy delivery  - Reputation for managing complicated deliveries  - Reputation among ASHAs | Rita, Gita and Sita are staff nurses at Community Health Centres in three different blocks. They have been working for a long time in their respective facilities, and have earned distinct reputation among the community they serve. A. Rita has earned the reputation of conducting deliveries very quickly. B. Gita has earned the reputation of managing complicated and risky deliveries. C. Sita has earned a good reputation among the ASHAs of the area. Who among the three staff nurses is likely to receive the most patients? | Do patients demand to be manged by one nurse or the other? On which criteria do they make this decision? What Role do ASHAs play in this? Do they have any preference?  At what point should oxytocin given to women, and for what reason?  We have also heard that sometimes oxytocin is given because the woman wants a fast delivery? Does that happen in your facility? Have you seen fellow nurses engage in such behavior? Why do you think they engage in the behavior?  Why do you think nurses want a fast delivery? How does it benefit the nurses? We have also heard that nurses get some kind of kickback after the use of oxytocin -- is that one of the reasons people use oxytocin in the wrong way? |
| S14: Promoting Handwash  - Social proof/Fixing broken windows  - Reminder and cues   - Risk perception | Rita, Gita and Sita are staff nurses at Community Health Centres in three different blocks. Their respective MOICs have implemented some actions to promote handwashing among the staff. A. Rita's MOIC has implemented a regular cleaning schedule for the entire facility which he monitors personally. He feels that if the surroundings are hygienic and well maintained, then people will remember to wash hands. B. Gita's MOIC has given each nurse a wristband with reminder for handwashing. He monitors that the nurses wear it during their duty. C. Sita's MOIC arranged special hygiene training for the entire staff. During this training, the focus was on the many different ways that not washing hands can affect mother and child health. Who among the three staff nurses is most likely to wash hands before delivery? | What are some things you often forget to do in your daily routine, and what helps you remember to do those things? Do you use these techniques at work?  What is the cleanliness protocol? What are the things in this SOP that are sometimes or often forgotten? Why do you think they are forgotten, and what would help you as reminders?  How do you think infections are spread in a facility?Who is responsible?At what time during delivery are infections spread? Before, during or after?  What can be done to stop the spread of infections? Handwashing? Infection prevention protocol? Clean birth place?  How long does it take the symptoms of an infection to appear after it has been spread?  Do you think not washing hands can lead to negative outcomes? Have you ever had this happen to you, can you tell us about a story you might have heard from other facilities?  How aware is the community and patients about infection prevention and detection? DO you tell them anything about this? How aware is the cleaning staff about the importance of hygiene and risks of infection? Do you tell them anything about this? |
| S15: Maintaining Clean Birth Place  - Teamwork/coordination with cleaners  - Risk perception  - Authoritative monitoring | Rita, Gita and Sita are staff nurses at Community Health Centres in three different blocks. Their respective MOICs have implemented some actions to maintain hygiene in the labour room.  A. Rita's MOIC holds regular meetings with nurses and cleaning staff to review status and resolve issues regarding maintenance of the labour room.  B. Gita's MOIC arranged special hygiene training for the entire staff. During this training, the focus was on the many different ways that unhygienic labour room can affect mother and child health.  C. Sita's MOIC visits the labour room twice everyday himself, and holds nurse and cleaning staff accountable for the condition of labour room.  Which nurse's labour room is most likely to be clean? | What is the cleanliness protocol? What are the things in this SOP that are sometimes or often forgotten? Why do you think they are forgotten, and what would help you as reminders?  How do you think infections are spread in a facility?Who is responsible?At what time during delivery are infections spread? Before, during or after?  What can be done to stop the spread of infections? Handwashing? Infection prevention protocol? Clean birth place?  How long does it take the symptoms of an infection to appear after it has been spread?  Do you think the labor room or maternity ward is a possible threat of infection in the newborn? How so? How can this be changed?  Do you think the cleaning staff does a good job in cleaning, do you think they should be given more responsibility? Who checks that the labor room and table is clean / sterlized and ready to be used for the next delivery? Does the cleaning staff do their job adequately or do you have to monitor and guide them? How often do you interact?  How aware is the cleaning staff about the importance of hygiene and risks of infection? Do you tell them anything about this? |
| S16: Reasons for low 48 hour stay  - No one to monitor and attend to patients  - No arrangements for attendant and ASHA to rest  - Too crowded and bad condition of maternity ward | Rita, Gita and Sita are staff nurses at Community Health Centres in three different blocks. They are discussing why 48 hour stay at their respective facilities is so low.  A. Rita says that the reason women leave is because the staff doesn't have enough time to monitor and attend to the patients.  B. Gita says that the reason women leave is because their ASHAs leave, as there are no facilities for ASHAs and they influence the women's decision the most.  C. Sita says that the reason women leave is that the condition of the facility, labour ward and other amenities are terrible, not convenient or comfortable at all.  Who among the three staff nurses is likely to have most women leave after delivery? | Do you think it's important for women to stay 48 hours or 24 hours? What can you do to make women and families stay for longer in the facility? What have you tried, what has worked? What has not worked? What did you to change your approach?  What are the reasons for the families wanting to rush after delivery? Who pushes the most - woman, relative, ASHA?  What kinds of complaints have you gotten from the families that did want to stay, but didn't have a positive experience?  What kind of complications and risks can arise during the first 2-48 hours after delivery? Do you inform the women/family about this? DO they understand? Is ASHA aware of these complications? |
| S17: Low Birth Weight  - If birth is normal, baby is out of danger  - Most babies are below the normal weight, but are healthy otherwise  - Smaller baby is preferable for an easier delivery | Rita, Gita and Sita are staff nurses at Community Health Centres in three different blocks. All three of them have just delivered newborns who are slightly under the ideal birth weight. They are considering whether special actions be taken to manage the respective low birth weight babies.  A. Rita feels that if the delivery is normal, without any complications, then the baby is out of danger.  B. Gita feels that most babies she delivers are slightly underweight, so as long as a baby is not very underweight, there is no danger.  C. Sita feels that low birth weight babies are actually better, because the delivery is much easier for smaller babies.  Who among the three staff nurses is least likely to conduct low birth weight baby procedures? | What are the most important things to check upon for a newborn baby? What do you think most nurses think are the best indicators of a healthy baby? What do you think are the most important vitals to check for the mother and the baby after delivery?  Is birth weight an important component?  Who is responsible for checking the baby’s birth weight and is it recorded? Where is it recorded?  What helps you in remembering all the things you have to do for the baby once it’s born?  What is the average birth weight? Are there complications even with the average birth weight? How often is there a complication with the weight?  What does the community think is a good enough baby birth weight? What about other nurses? What do you think is a good birth weight for a baby?  What kind of complications does low birth weight cause? Can it be harmful to the baby? |

SET 1

**1.** जया, दीपा और शिवानी तीन अलग-अलग ब्‍लॉकों के CHCs में तीन स्‍टाफ नर्स हैं। उनमें से प्रत्‍येक के अस्‍पताल में एक गर्भवती महिला आती है और उन्‍हें इन हालातों का सामना करना पड़ता है:

**1.1** मरीज का BP बहुत ज्‍यादा है और नर्सें यह सोचविचार कर रही हैं कि क्‍या उसे ज़िला अस्‍पताल में रेफर किया जाए या CHC में डिलीवरी करवाई जाए।

1. जया को लगता है कि मुश्किल डिलीवरी करवाने से उसे अपनी बस्‍ती में और ASHAs के बीच आदर मिलेगा और उसकी इज्जत बढ़ेगी।
2. दीपा को पूरा विश्‍वास है कि वो प्रकट हो सकने वाली मुश्किल को संभाल लेगी। पहले भी उसने ऐसे कई केसिस को सँभाला है और बिना किसी मुश्किल के सफलतापूर्वक डिलीवरी करवाई हैं।
3. शिवानी को लगता है कि मरीज को ज़िला अस्‍पताल रेफर करना बेकार है क्‍योंकि परिवार वाले ज़ि‍ला अस्‍पताल जाने का हमेशा विरोध करते हैं, और मांग करते हैं कि CHC में ही डिलीवरी करवाई जाए।

इन तीन स्‍टाफ नर्सों में से किसके द्वारा मरीज की CHC में डिलीवरी करवाने और ज़िला अस्‍पताल में रेफर नहीं करने की सबसे ज्‍यादा संभावना है?

गर्भवती महिला को प्रतिष्‍ठान में भर्ती करने का क्‍या प्रोटोकॉल है? वह पहले कहां जाती है?

अपने प्रतिष्‍ठान में एक ठेठ प्रसव-पूर्व जांच के बारे में हमें बताइये।

- क्‍या? कौन-कौन से पैरामीटरों की जांच की जाती है? मेडिकल हिस्‍ट्री?

- कहां? क्‍या यह जगह बदलती रहती है या एक फिक्‍स्‍ड जगह है?

- उसके महत्‍वपूर्ण अंगों की जांच कौन करता है? कौन इंचार्ज है?

- अगर कोई कॉम्‍प्‍लीकैशन है तो मरीज को रेफर करने का फैसला कौन करता है?

- यह फैसला कैसे किया जाता है? यह फैसला करने के लिए कौन सा क्राइटेरिया इस्‍तेमाल किया जाता है?

- मरीज को बाहर रेफर करने से पहले, कौन से इंतजाम करने होते हैं?

- कहां और कौन से वाइटल्‍स रिकॉर्ड किए जाते हैं? किसके द्वारा? कब और कैसे आप डिलीवरी के दौरान इन रिकॉर्डों को इस्‍तेमाल करते हैं?

- प्रसव-पूर्व जांचों को करने और HRPs को रेफर करने में आपको किस तरह की दिक्‍कतों का सामना करना पड़ता है? आप इन दिक्‍कतों पर कैसे काबू पाती हैं? उदाहरण बताइये।

आपके पास एक हफ्ते में कितने HRP केसिस आते हैं? उनमें से कितने मॉडरेट और कितने हाई रिस्‍क होते हैं? आप कौन से केसिस को सबसे ज्‍यादा देखती हैं – प्रीमैच्‍योरिटी, एनीमिया, ईक्‍लैम्‍पसिया? आप किस तरह के HRPs को सबसे ज्‍यादा रेफर करती हैं? आप किस HRP कंडीशन को आराम से संभाल सकती हैं?

क्‍या मरीज/परिवार प्रतिष्‍ठान से बाहर रेफर किए जाने के खिलाफ होते हैं? क्‍यों? आप ऐसे मरीजों को कैसे संभालती हैं?

क्‍या इससे रेफरल प्रोसेस पर किसी तरह से असर पड़ता है? कॉम्‍प्‍लीकेटिड डिलीवरी के बारे में परिवार वाले कैसे रिएक्‍शन करते हैं? यदि कोई कॉम्‍पलीकेशन पैदा होता है तो नर्स के लिए परिवार का क्‍या रिएक्‍शन होता है?

आपके ख्‍याल से कौन सी मुख्‍य वजह है जिसके कारण दूसरी नर्सें HRP केसिस को कम रेफर करती हैं?

**1.2** तीनों नर्सों ने अपने अस्‍पताल में डिलीवरी करवाने का फैसला किया। लेकिन डिलीवरी के दौरान मरीज को ईक्‍लैम्‍पसिया की तकलीफ हो गई। नर्सों को जल्‍दी से फैसला करना है कि इस मुश्किल को कैसे सँभाला जाए।

A. जया तुरंत इस केस को ज़िला अस्‍पताल रेफर करती है, क्‍योंकि उसको लगता है कि ऐसे मुश्किल केस को CHC में नहीं सँभाला जा सकता है।

B. दीपा ऐसी मुश्किल को संभालने का सही प्रक्रिया पता लगाने के लिए केस शीट इस्‍तेमाल करती है, क्‍योंकि उसे लगता है कि इसे जल्‍दी से जल्‍दी से सँभाला जाना है ताकि आगे और ज्‍यादा मुश्किल से बचा जा सके।

C. शिवानी LMO से मदद मांगती है क्‍योंकि उसे लगता है कि उसे ऐसी मुश्किल को संभालने के लिए मदद और समर्थन की जरूरत है।

इस मुश्किल को संभालने में किस स्‍टाफ नर्स की कार्यवाही के सफल होने की सबसे ज्‍यादा संभावना है?

डिलीवरी के दौरान, आपको किस तरह की कॉम्‍प्‍लीकैशन का सामना करना पड़ता है? जब डिलीवरी के दौरान कोई कॉम्‍प्‍लीकैशन पैदा होती है तो उसको संभालना किसका काम है? जब कॉम्‍प्‍लीकैशन्‍स पैदा होती हैं तो आप उन्‍हें कैसे संभालती हैं? (LMO या नर्स मेंटॉर को बुलाती हैं, क्‍या वे अपने से संभालने की कोशिश करते हैं, अगर वे अपने से संभालने की कोशिश करते हैं तो वे किन सहयोगियों की मदद लेते हैं?)

अगर आपको आपके LMO/NM से मदद नहीं मिल सकती है तो आप कॉम्‍प्‍लीकैशन को कैसे संभालती हैं? क्‍या आपको कॉम्‍प्‍लीकैशन्‍स को संभालने पूरा आत्‍मविश्‍वास महसूस होता है? हमें एक ऐसा केस बताइये जिसमें आपको खुद कॉम्‍प्‍लीकैशन संभालने में आत्‍मविश्‍वास महसूस नहीं हुआ था और आपने उस केस में क्‍या किया था? तब आपने कौन से मौजूदा सहयोगियों से मदद ली थी? कॉम्‍प्‍लीकैशन की रोकथाम करने के लिए क्‍या किया जा सका था?

क्‍या कॉम्‍पलीकेटिड या अनजान केसिस को संभालने में केस शीट से मदद मिलती है?

बिना किसी मदद के किसी कॉम्‍प्‍लीकैशन को संभालने के बारे में आपको कैसा महसूस हुआ था? जब आप वह कर रही थीं तब आपके मन क्‍या विचार आ रहे थे? वहां मौजूद टीम मेम्‍बर्स में से, आपने किससे मदद ली थी और कैसे?

हमें ऐसे वक्‍त का एक उदाहरण बताइये जब किसी कॉम्‍प्‍लीकैशन में LMO ने आपकी मदद की थी? आपने LMO से कैसे संपर्क किया था?

**1.3** नर्सें मां को बचाने में सफल हुईं। बदकिस्‍मती से, नर्सें बच्‍चे को नहीं बचा पाईं जिसे बर्थ ऐस्फिक्सिया की तकलीफ थी। इस हादसे से उन्‍हें परेशान कर दिया और उसके बाद से वे लगातार इस बारे में सोच रही हैं।

A. जया को लगता है कि अस्‍पताल में कर्मचारियों की कमी है और उसे दी गई ट्रेनिंग भी नाकाफी है। डिलीवरी करवाने में मदद और कौशलों की कमी के कारण, ऐसे हादसों का होना निश्चित है।

B. दीपा को लगता है कि उसने अस्‍पताल में सैकड़ों डिलीवरी करवाई हैं, लेकिन केवल कुछ में ही बच्‍चे की मौत हुई है। सफल डिलीवरी की संख्‍या को देखते हुए, कुछ-हादसे ज्‍यादा खराब नहीं है।

C. शिवानी को लगता है कि ऐसे हादसों के लिए परिवार वालों और ASHA का बर्ताव एवं फैसला जिम्‍मेदार है। उन्‍हें अनेक प्रक्रियाओं और कार्यों की अहमियत का अहसास नहीं है, मसलन: अगर ANC ठीक से की जाए तो ऐसे हादसों की रोकथाम हो सकती है।

इनमें से किस सोच से तसल्‍ली और राहत मिलने की संभावना सबसे ज्‍यादा है?

बच्‍चा पैदा होने के बाद, पहले कुछ घंटों के अंदर नवजन्‍मे बच्‍चे के लिए सबसे बड़ा खतरा क्‍या होता है?

आपके पास एक हफ्ते में दम घुटने के कितने केसिस आते हैं? क्‍या आपको जन्‍म के समय दम घुटने के केसिस को संभालने में आत्‍मविश्‍वास महसूस होता है? दम घुटने के केस को संभालने का सबसे मुश्किल हिस्‍सा क्‍या है?

वह कौन से मुख्‍य कारण हैं जिनकी वजह से कॉम्‍प्‍लीकैशन के फलस्‍वरूप नैगेटिव नतीजे मिलते हैं? आपको क्‍यों लगता है कि यह कारण है? इसकी रोकथाम करने में क्‍या मदद कर सकता है?

हमें खराब नतीजे का उदाहरण बताइये। आपके ख्‍याल से यह किस वजह से हुआ था? आमतौर पर, इस केस में, इसको संभालने की मानक कार्यपद्धति (SOP) क्‍या है, और इस बार वह कामयाब क्‍यों नहीं रही? इसकी रोकथाम करने के लिए क्‍या किया जा सकता था?

क्‍या परिवार आपकी सलाह को मानते हैं – 10 महिलाओं में से कितनी आपकी बात सुनती हैं? यदि नहीं तो क्‍या उन्‍हें आपकी बात सुनवाने में ASHA आपकी मदद करती है? और कौन उनसे आपके साथ सहयोग करवा सकता है? परिवार वाले किसी बात सबसे ज्‍यादा सुनते हैं: ASHA, आप, या कोई सीनियर?

क्‍या आपको कॉम्‍प्‍लीकैशन्‍स संभालने में आत्‍मविश्‍वास महसूस होता है? क्‍या आपको लगता है कि किसी प्रोसीजर के बारे में आपको ज्‍यादा ट्रेनिंग या मदद की जरूरत है?

SET 2

**2.** सुषमा, निधि और सुमन तीन अलग-अलग ब्‍लॉकों के CHCs में तीन स्‍टाफ नर्स हैं। उन सबने अभी-कभी एक डिलीवरी करवाई है और अब उनके सामने ये हालात हैं।

**2.1** वे नाल हटाने का काम दाई के जिम्‍मे छोड़कर अगले मरीज के पास जाने की सोच रही हैं।

A. सुषमा को लगता है कि सफल डिलीवरी के बाद जच्‍चा-बच्‍चा की सेहत के लिए बड़ा खतरा खत्‍म हो जाता है, और AMTSL एक आसान और खतरे से मुक्‍त काम है जो दाई आसानी से कर सकती है।

B. निधि को मुश्किल प्रक्रिया और हाई रिस्‍क के कारण डिलीवरी के तुरंत बाद काफी थकान महसूस होती है, इसलिए वो डिलीवरी के तुरंत बाद के काम दाई को सौंपना पसंद करती है।

C. सुमन को लगता है कि नर्स की सबसे जरूरी जिम्‍मेदारी डिलीवरी है, और कम जरूरी कार्यों में उनकी मदद करने के लिए सहायक कर्मचारी वहां मौजूद हैं।

इन तीन स्‍टाफ नर्सों में किसके द्वारा नाल हटाने/AMTSL का काम दाई को सौंपे जाने की संभावना सबसे ज्‍यादा है?

जब महिला प्रतिष्‍ठान में होती है तब कौन समय सबसे महत्‍वपूर्ण है? तनावपूर्ण? कौन सा सबसे ज्‍यादा राहत देने वाला है? डिलीवरी के बाद, किस चीज की तुरंत देखभाल करना सबसे ज्‍यादा जरूरी होता है?

डिलीवरी के दौरान और डिलीवरी के बाद के प्रोसेस में आपकी क्‍या जिम्‍मेदारियां हैं? किन जिम्‍मेदारियों में दाई आपकी मदद करती है? किन जिम्‍मेदारियों के बारे में आपको भरोसा है कि दाई उन्‍हें खुद संभाल लेगी?

किस वक्‍त पर आपको लगता है कि नवजन्‍मा बच्‍चा महफूज और खतरे से बाहर है? मां के बारे में किस वक्‍त?

हमें विस्‍तार से बताइये कि बच्‍चा पैदा होने के ठीक बाद क्‍या होता है? आप कैसे याद करती हैं कि आपको डिलीवरी के बाद किन चीजों पर ध्‍यान देना है?

क्‍या वहां कोई चेक-लिस्‍ट है जिसे आप डिलीवरी के बाद के प्रोसीजर के लिए देखती हैं? आप उसे किस वक्‍त इस्‍तेमाल करती हैं?

क्‍या आपको लगता है कि डिलीवरी के बाद के प्रोसीजर्स जैसे थर्मल केयर, स्‍तनपान के लिए कम प्रेरणा है? किस वजह से यह होता है?

क्‍या आपको नाल हटाने में कोई कॉम्‍पलीकेशन होती है? आप उन्‍हें कैसे संभालती हैं? क्‍या आपके बिना नाल को हटा सकती है?

**2.2** वे पाती हैं कि नवजन्‍मे बच्‍चे का वज़न आदर्श वज़न से कम है। वे सोचविचार कर रही हैं कि क्‍या कम वज़न वाले बच्‍चे को संभालने के लिए खास काम किए जाने की जरूरत है।

A. सुषमा को लगता है कि अगर किसी मुश्किल के बिना नॉर्मल डिलीवरी हुई है तो बच्‍चा खतरे से बाहर है।

B. निधि को लगता है कि उसके द्वारा डिलीवरी करवाए गए ज्‍यादातर बच्‍चों को थोड़ा कम वज़न रहा है इसलिए जब तक बच्‍चे का वज़न बहुत ज्‍यादा कम नहीं है तब तक कोई खतरा नहीं है।

C. सुमन को लगता है कि कम वज़न वाले बच्‍चे वास्‍तव में बेहतर होते हैं क्‍योंकि छोटे बच्‍चों की डिलीवरी करवाना ज्‍यादा आसान होता है।

इन तीन स्‍टाफ नर्सों में किसके द्वारा कम वज़न वाले बच्‍चे की प्रक्रियाओं को पूरा किए जाने की संभावना सबसे ज्‍यादा है?

एक नवजन्‍मे बच्‍चे के लिए किन बातों की जांच करना सबसे ज्‍यादा जरूरी काम है? आपके ख्‍याल से, ज्‍यादातर नर्सें किन संकेतों को एक स्‍वस्‍थ बच्‍चे का सर्वश्रेष्‍ठ संकेत मानती हैं? आपके ख्‍याल से, डिलीवरी के बाद मां और बच्‍चे के शरीर के किन जरूरी अंगों की जांच करना सबसे ज्‍यादा जरूरी है?

क्‍या पैदा होने पर वज़न भी एक महत्‍वूपूर्ण चीज है?

बच्‍चे के पैदा होने पर उसका वज़न देखना किसकी जिम्‍मेदारी है और क्‍या इसे रिकॉर्ड किया जाता है? इसे कहां रिकॉर्ड किया जाता है?

बच्‍चा पैदा होने पर आपको कौन-कौन से काम करने हैं, यह याद करने में कौन सी चीज/बात आपकी मदद करती है?

बच्‍चों के पैदा होने पर, औसत वज़न कितना है? क्‍या वहां बच्‍चों के पैदा होने पर औसत वज़न के बारे में भी कोई कॉम्‍प्‍लीकैशन्‍स हैं? कितनी बार वहां वज़न के बारे में कोई कॉम्‍प्‍लीकैशन होती है?

समुदाय के ख्‍याल से नवजन्‍मे बच्‍चे का कितना वज़न अच्‍छा पर्याप्‍त है? दूसरी नर्सों का क्‍या ख्‍याल है? आपके ख्‍याल से नवजन्‍मे बच्‍चे का कितना वज़न अच्‍छा है?

पैदा होने पर कम वज़न किस तरह के कॉम्‍प्‍लीकैशन्‍स पैदा करता है? क्‍या यह बच्‍चे के लिए नुकसानदायक हो सकता है?

**2.3** डिलीवरी के बाद, उन्‍हें परिवार वालों के कुछ ऐसे कार्य दिखाई देते हैं जिनसे उन्‍हें गुस्‍सा आता है।

A. सुषमा देखती है कि महिला की जेठानी नवजन्‍मे बच्‍चे को कुछ खिला रही है।

B. निधि देखती है कि महिला की सास नाल पर तेल लगा रही है।

C. सुमन देखती है कि महिला का पति डिलीवरी के तुरंत बाद नवजन्‍मे बच्‍चे को उससे दूर ले गया।

इन तीन स्‍टाफ नर्सों में किसके द्वारा परिवार वालों के इन कार्यों पर गुस्‍सा होने की सबसे ज्‍यादा संभावना है?

परिवार को सबसे बदतर बर्ताव कौन से हैं? आमतौर पर, सबसे ज्‍यादा देखे गए प्रतिकूल बर्ताव कौन से हैं?

क्‍या आपको इनमें से किसी बर्ताव और धारणा में कोई सार्थकता दिखाई देती है? आपके ख्‍याल से, ये धारणाएं समुदाय के लिए कैसे फायदेमंद हैं?

आप किन मुख्‍य विषयों के बारे में महिलाओं को सलाह-मशविरा देती हैं? किन विषयों के बारे में, आपको परिवार वालों से सबसे ज्‍यादा विरोध महसूस होता है?

आप यह पक्‍का करने के लिए क्‍या कर सकती हैं कि महिला को अधिक जानकारी हो और एक अच्‍छा निर्णय लेने के लिए उसके पास पर्याप्‍त जानकारी हो?

डिलीवरी के बाद के प्रोसीजर में, मरीज और उसके परिवार वाले किन तरीकों से सहयोग करते हैं?

- क्‍या आपको इस प्रोटोकॉल के बारे में परिवारों से विरोध का सामना करना पड़ता है? किसलिए? आप ऐसे हालातों में कैसे प्रतिक्रिया करती हैं?

- महिलाओं द्वारा निर्देशों का पालन नहीं किए जाने की मुख्‍य वजहें क्‍या हैं?

- थर्मल-केयर और स्‍तनपान के मौजूदा कायदे बारे में आपका क्‍या सोचना है? क्‍या वहां कोई सामुदायिक कायदा है जो आपके ख्‍याल से उपयोगी और फायदेमंद है?

**2.4** इन तीनों को अपने काम में कुछ दिक्‍कतों का सामना करना पड़ रहा है:

A. सुषमा के अस्‍पताल में कर्मचारियों की कमी है, और इसलिए उसे लंबी शिफ्ट में काम करना पड़ता है। उसकी शिफ्ट बदलती भी रहती है, और उसे अपने काम में बहुत कम या बिल्‍कुल मदद नहीं मिलती है।

B. निधि को अक्‍सर मरीज के परिवार वालों की अनुचित मांगों और खराब बर्ताव का सामना करना पड़ता है, जो खतरे और प्रक्रिया को नहीं समझते हैं और लेबर रूम के चारों ओर परेशानी एवं टेंशन पैदा करते हैं।

C. सुमन के अस्‍पताल में ज़ि‍ला के अफसर जांच करने के लिए लगातार आते रहते हैं और अस्‍पताल के कार्य की जिम्‍मेदारी अक्‍सर उस पर आ जाती है। उसे टारगेट नहीं पूरे करने के लिए, उसे अस्‍पताल में अपने सीनियर्स की अक्‍सर डांट खानी पड़ती है।

इन तीन स्‍टाफ नर्सों में किसको काम में दिक्‍कत के कारण सबसे ज्‍यादा तनाव महसूस होने की संभावना है?

- क्‍या आप किसी ऐसे समय के बारे में विस्‍तार से बता सकती हैं जब आप तनाव में थीं? यह तनाव कैसे पैदा हुआ था, यह किस वजह से हुआ था, किन-‍किन बातों ने इसमें योगदान दिया था? आप इस पर कैसे काबू पाती हैं? किन चीजों ने तनाव पर काबू पाने में आपकी मदद की? क्‍या तनाव से आपके कामकाज और परफोरमेंस पर असर पड़ा था?
- सबसे जरूरी टारगेट कौन से हैं? किन टारगेटों को हासिल करना मुश्किल है और क्‍यों? ये टारगेट आपको कैसे सूचित किए जाते हैं? टारगेटों की तुलना में आपकी परफोरमेंस पर कौन नजर रखता है?
- मरीजों के कौन से बर्तावों और मांगों से आप पर सबसे ज्‍यादा जोर पड़ता है और सबसे ज्‍यादा चिढ़ होती है? आप अपने प्रतिष्‍ठान में आने वाले मुश्किल मरीजों और उनके परिवारों को कैसे संभालती हैं? आप उनकी उम्‍मीदों को कैसे संभालती हैं?
- आप मरीजों और उनके परिवारों के साथ तब कैसा संबंध बनाकर रखती हैं जब वह प्रतिष्‍ठान में होते हैं? (क्‍या वे कम से कम बातचीत करते हैं, क्‍या वे दोस्‍ताना बर्ताव करते हैं, क्‍या वे विनम्र होते हैं और क्‍या ऐसे मामलों में मरीजों ने उनका फायदा उठाया है?)
- क्‍या आपकी शिफ्ट फिक्‍स्‍ड है या बदलती रहती है? आप अपने भारी वर्कलोड और लंबी शिफ्ट को कैसे संभालती हैं?

अत्‍यधिक तनावपूर्ण हालातों में आपको किससे सबसे ज्‍यादा मदद मिलती है?

SET 3

**3.** रामा, नीलम और शीतल तीन अलग-अलग ब्‍लॉकों के CHCs में तीन स्‍टाफ नर्स हैं। उनके बीच इन बातों पर चर्चा चल रही है:

**3.1** केस शीट का इस्‍तेमाल और अहमियत

A. रामा कहती है कि केस शीट बहुत फायदेमंद है क्‍योंकि अगर आप इसे हर बार भरती हैं तो बाद में मां या बच्‍चे के स्‍वास्‍थ्‍य में किसी मुश्किल के लिए आपको दोष नहीं दिया जा सकता है।

B. नीलम कहती है कि केस शीट का सबसे अच्‍छा फायदा ऐसी मामूली लेकिन जरूरी बातों का याद रख पाना है जो आप ध्‍यान बंटने और दबाव के कारण डिलीवरी के दौरान भूल सकती हैं।

C. शीतल का कहना है कि केस शीट मुश्किल केसिस में सबसे ज्‍यादा फायदेमंद है जिन्‍हें आपने पहले देखा या सँभाला नहीं है। ऐसे केसिस में, केस शीट आपको बता सकती है कि क्‍या करना है।

इन तीन स्‍टाफ नर्सों में किसके द्वारा केस शीट को सबसे ज्‍यादा इस्‍तेमाल किए जाने की संभावना है?

क्‍या आपको लगता है कि केस शीट एक जरूरी दस्‍तावेज है? क्‍यों?

आपके ख्‍याल से, प्रतिष्‍ठान में केस शीट क्‍यों शुरु की गई थी?

क्‍या आपने केस शीट की उपयोगिता को बेहतर बनाने के लिए कुछ किया है? आपके पास कौन से नुक्‍ते हैं, क्‍या आपने इनके बारे में प्रतिष्‍ठान को बताया है? उनका रिएक्‍शन कैसा रहा है?

जब आप केस शीट नहीं भरती हैं तब क्‍या होता है / क्‍या नतीजा रहता है?

क्‍या आप ऐसा कोई उदाहरण बता सकती हैं जब केस शीट को नहीं भरा गया था और कुछ नहीं हुआ? क्‍या आप किसी ऐसे वक्‍त के बारे में बता सकती हैं जब किसी केस को संभालने में केस शीट ने आपकी मदद की हो?

क्‍या केस शीट परिवारों के प्रतिकूल और असहयोगी रिवाजों को रिकॉर्ड करने में आपकी मदद करती है?

**3.2** वे बातचीत कर रही हैं कि नर्स मेंटॉर उनकी कैसे मदद करती हैं और उनके कार्य में सुधार लाती हैं।

A. रामा कहती है कि उसे जो शिक्षा और ट्रेनिंग मिली थी, उसके आगे मां-बच्‍चे की सेहत के बारे में उसकी जानकारी को बेहतर बनाने लाने और नए कौशल विकसित करने में नर्स मेंटॉर ने उसकी मदद की।

B. नीलम कहती है कि मुश्किल केसिस और नई तरह के केसिस को संभालने में नर्स मेंटॉर ने उसकी मदद की जिन केसिस को संभालने में वह असमर्थ/अनिच्‍छुक थी या संभालते समय गलतियां कर देती थीं।

C. शीतल कहती है कि नर्स मेंटॉर ने उसकी जरूरतों और दिक्‍कतों को समझकर मदद की है, और उनमें से कुछ दिक्‍कतों को सुलझाने के लिए उनको उपयुक्‍त अफसरों और कर्मचारियों के सामने उठाया है।

इन तीन स्‍टाफ नर्सों में किसके द्वारा उसकी नर्स मेंटॉर की सबसे ज्‍यादा कदर किए जाने की संभावना है?

कौन आपकी टीम का हिस्‍सा है? उनके क्‍या-क्‍या कार्य हैं?

जब आपको मदद की जरूरत होती है तब कौन आपकी सबसे ज्‍यादा मदद करता है?

आपके ख्‍याल से, कार्य के किन क्षेत्रों में टीम वर्क कामों को ज्‍यादा सहज और बेहतर बना सकता है?

कार्य के किन क्षेत्रों में, आपको लगता है कि आपको मदद नहीं मिल रही है, और उसे आपके लिए बेहतर बनाने के लिए क्‍या किया जा सकता है?

कार्य के कौन से क्षेत्र एक मजबूत टीम होने के कारण पहले से ही अच्‍छा काम कर रहे हैं?

हमें ऐसे वक्‍त का एक उदाहरण बताइये जब नर्स मेंटॉर किसी केस में फायदेमंद और मददगार साबित हुई हो? हमें एक उदाहरण बताइये जब नर्स मेंटॉर मदद कर सकती थी लेकिन उसने नहीं की। नर्स मेंटॉर की वजह से आपके किन कौशलों सबसे ज्‍यादा सुधार आया है? किन कौशलों में, आपको ज्‍यादा मदद की जरूरत नहीं है?

आप किन केसिस में मदद के लिए MO या LMO के पास जाती हैं? किन केसिस में, आप मदद के लिए नर्स मेंटॉर के पास जाती हैं?

**3.3** केस शीट में कुछ बदलाव किए गए हैं।

A. रामा के अस्‍पताल में, पाठ्यसामग्री को कम करके और चित्र/ग्राफिक शामिल करके केस शीट को समझना ज्‍यादा आसान बनाया गया है।

B. नीलम के अस्‍पताल में, केवल सबसे जरूरी जानकारी और कार्यों को फोकस करके, और बाकी को केस शीट से हटाकर केस शीट को छोटा बनाया गया है।

C. शीतल के अस्‍पताल में, केस शीट को तीन छोटी शीटों में बांट दिया गया है, एक को डिलीवरी से पहले, दूसरी को डिलीवरी के बाद, और तीसरी को अस्‍पताल से छुट्टी होने पर भरना है, ताकि नर्स को इन्‍हें सही वक्‍त पर इस्‍तेमाल करना याद रहे।

इन तीन स्‍टाफ नर्सों में किसके द्वारा इन बदलावों का समर्थन करने और केस शीट को ज्‍यादा इस्‍तेमाल करना शुरु करने की सबसे ज्‍यादा संभावना है?

आप एक दिन में कितनी केस शीट भरती हैं? क्‍या आप डिलीवरी के दौरान, डिलीवरी के तुरंत बाद या दिन खत्‍म होने पर केस शीट भरती हैं? क्‍या केस शीट को भरना आपके वर्कलोड को बढ़ाता है?

केस शीट में सबसे ज्‍यादा उपयोग हिस्‍से कौन से हैं? केस शीट के सबसे कम उपयोगी हिस्‍से कौन से हैं?

क्‍या केस शीट भरते वक्‍त आपको किसी मदद या सहायता की जरूरत होती है? क्‍या आप केस शीट को पूरी तरह से समझती हैं?

क्‍या आपको केस शीट भरना हमेशा याद रहता है, या अक्‍सर आपको याद दिलाने की जरूरत होती है?

SET 4

**4.** शोभा, ऊषा और दीपा तीन अलग-अलग ब्‍लॉकों के CHCs में तीन स्‍टाफ नर्स हैं। वे सब सोच रहीं है कि वे अपने कार्य को कैसे बेहतर बना सकती हैं।

**4.1** वे सोच रही हैं कि क्‍या उन्‍हें बच्‍चों के बीच अंतर रखने और गर्भनिरोधक उपायों के बारे में गर्भवती महिलाओं को सलाह देने में ज्‍यादा वक्‍त लगाना चाहिए लेकिन उनके मन में कुछ शंकाएं हैं।

A. शोभा को लगता है कि परिवार नियोजन के बारे में ऐसी सलाह देना ASHA की जिम्‍मेदारी है, और उन्‍हें इसके लिए पैसे तक मिलता है।

B. ऊषा को लगता है कि सलाह देना बेकार है क्‍योंकि परिवार नियोजन के बारे में परिवार वालों अपनी अपनी धारणाएं और कसौटी होती है, और वे इस बारे में डाक्‍टरों और नर्सों की बात नहीं सुनते हैं।

C. दीपा को लगता है कि चूँकि डिलीवरी के वक्‍त परिवार वाले हमेशा जल्‍दी और तनाव में होते हैं, और उसके बाद उनका ध्‍यान बहुत ज्‍यादा बंटा होता है और थके हुए होते हैं, इसलिए यह सलाह देने का सही वक्‍त और जगह नहीं है।

इन तीन स्‍टाफ नर्सों में किसके द्वारा परिवार नियोजन की सलाह देने पर ज्‍यादा समय लगाने की सबसे कम संभावना है?

हमें अपने किसी काउंसलिंग सैशन का उदाहरण बताइये, और वह कैसा रहा था? क्‍या कामयाब हुआ था और क्‍या नहीं? वह क्‍यों कामयाब नहीं हुआ था? आपके ख्‍याल से किसे कांउसलिंग करनी चाहिए? क्‍यों --> क्‍या वे उनकी बात सुनते हैं?

आप किन मुख्‍य विषयों के बारे में महिलाओं को सलाह-मशविरा देती हैं? किन विषयों के बारे में, आपको परिवार वालों से सबसे ज्‍यादा विरोध महसूस होता है?

आप यह पक्‍का करने के लिए क्‍या कर सकती हैं कि महिला को अधिक जानकारी हो और एक अच्‍छा निर्णय लेने के लिए उसके पास पर्याप्‍त जानकारी हो?

आप एक हफ्ते में कितने लोगों का सलाह-मशविरा देती हैं? कितनी डिलीवरी? कब आप महिला को सलाह-मशविरा देती हैं? आपके ख्‍याल से, महिला को सलाह-मशविरा देने के लिए कौन सा वक्‍त सही है?

हमें एक उदाहरण बताइये जब आपने किसी परिवार को यकीन दिलाने के बड़ी कोशिश की हो और वह कामयाब हुई हो? आमतौर पर, महिलाओं को सलाह-मशविरा देते वक्‍त आपको कौन सी सबसे बड़ी चुनौतियों का सामना करना पड़ता है? परिवार वाले? इसने सलाह-मशविरा देने के आपके तरीके को कैसे बदला है?

परिवार नियोजन की सलाह के बारे में, मरीज कितनी बात सुनते हैं? परिवार नियोजन का सलाह-मशविरा देने के लिए, परिवार में कौन सा व्‍यक्ति सर्वश्रेष्‍ठ रहता है? क्‍या वे इस बारे में ASHA की या आपकी बात सुनते हैं? कौन सी धारणाएं और रवैये लोगों से परिवार नियोजन का विरोध करवाते हैं?

**4.2** उनके LMOs ने लेबर रूप में स्‍वच्‍छता बनाए रखने के लिए कुछ कार्य करवाए हैं।

A. शोभा की LMO लेबर रूम की स्थिति को रिव्‍यू करने और रखरखाव के मसलों को सुलझाने के लिए नर्सों और सफाई कर्मचारियों के साथ नियमित रूप से बैठक करती है।

B. ऊषा की LMO ने सभी कर्मचारियों के लिए विशेष ट्रेनिंग का इंतजाम किया है। इस ट्रेनिंग में ऐसे अनेक तरीकों पर फोकस किया गया था जिनसे अस्‍वच्‍छ लेबर रूम मां-बच्‍चे के स्‍वास्‍थ्‍य को प्रभावित कर सकता है।

C. दीपा की LMO हर दिन दो बार खुद लेबर रूम का दौरा करता है, लेबर रूम की हालत के लिए नर्सों और सफाई कर्मचारियों को जिम्‍मेदार ठहराता है।

किस नर्स का लेबर रूम साफ रहने की सबसे ज्‍यादा संभावना है?

स्‍वच्‍छता का क्‍या प्रोटोकॉल है? इस मानक कार्यपद्धति (SOP) ऐसे कौन से काम हैं जो कभी-कभी या अक्‍सर भूल जाते हैं? आपके ख्‍याल से, वे क्‍यों भूल जाते हैं, और उन्‍हें याद दिलाने में कौन सी चीज/काम आपकी मदद करेगा?

आपके ख्‍याल से, किसी प्रतिष्‍ठान में इंफेक्‍शन क्‍यों फैलता है? इसके लिए कौन जिम्‍मेदार है? डिलीवरी के दौरान, किस वक्‍त इंफेक्‍शन फैलते हैं? पहले, उसके दौरान या बाद में?

इंफेक्‍शन को फैलने से रोकने के लिए क्‍या किया जा सकता है? हाथ धोना? इंफेक्‍शन की रोकथाम करने का प्रोटोकॉल? बच्‍चा पैदा होने की जगह को साफ करना?

इंफेक्‍शन फैल जाने के बाद, उसके लक्षण दिखाई देने में कितना वक्‍त लगता है?

क्‍या आपको लगता है कि लेबर रूम या मेटरनिटी वार्ड नवजन्‍मे बच्‍चे के लिए इंफेक्‍शन का एक संभावित खतरा है? कैसे? इसे कैसे बदला जा सकता है?

क्‍या आपको लगता है कि सफाई कर्मचारियों ने सफाई करने को अच्‍छा काम किया है, क्‍या आपको लगता है कि उन्‍हें ज्‍यादा जिम्‍मेदारियां सौंपी जानी चाहिए? यह कौन देखता है कि लेबर रूम और टेबल साफ/स्‍टर्लाइज्‍ड है और अगली डिलीवरी के लिए तैयार है? क्‍या सफाई कर्मचारी अपने काम को काफी अच्‍छे से करते हैं या आपको उन पर नजर रखनी और उनका मार्गदर्शन करना होता है? कितनी बार आपकी आपस में बातचीत होती है?

स्‍वच्‍छता की अहमियत और इंफेक्‍शन के खतरे के बारे में सफाई करने वाले कर्मचारियों को कितनी जानकारी है? क्‍या आप उन्‍हें इस बारे में कुछ बताती हैं?

**4.3** उनके MOIC ने कर्मचारियों के बीच हाथ धोने के रिवाज को बढ़ावा देने के लिए कुछ कार्य किए हैं।

A. शोभा के MOIC पूरे अस्‍पताल के लिए एक नियमित सफाई कार्यक्रम लागू किया है जिसे वह खुद मॉनीटर करता है। उसे लगता है कि अगर चारों ओर साफ-सुथरा और अच्‍छा रखरखाव है तो लोग हाथ धोना याद रखेंगे।

B. ऊषा के MOIC ने हर-एक नर्स को हाथ धोने के बारे में याद दिलाने वाला एक रिस्‍टबैंड दिया है। वह मॉनीटर करता है कि नर्सें ड्यूटी के दौरान इसे पहनें।

C. दीपा के MOIC सारे स्‍टाफ के लिए विशेष स्‍वच्‍छता ट्रेनिंग का इंतजाम किया है। इस ट्रेनिंग में उन अनेक तरीकों पर फोकस किया गया था जिनसे हाथ नहीं धोने पर मां-बच्‍चे का स्‍वास्‍थ्‍य प्रभावित हो सकता है।

इन तीन स्‍टाफ नर्सों में किसके द्वारा डिलीवरी के पहले हाथ धोए जाने की सबसे ज्‍यादा संभावना है?

वे कौन से कुछ-एक काम हैं जिन्‍हें करना आप अपनी रोजचर्या में अक्‍सर भूल जाती हैं, और उन कामों का करना याद रखने में आपको किससे मदद मिलती है? क्‍या आप अपने काम में इन तकनीकों को इस्‍तेमाल करती हैं?

स्‍वच्‍छता का क्‍या प्रोटोकॉल है? इस मानक कार्यपद्धति (SOP) ऐसे कौन से काम हैं जो कभी-कभी या अक्‍सर भूल जाते हैं? आपके ख्‍याल से, वे क्‍यों भूल जाते हैं, और उन्‍हें याद दिलाने में कौन सी चीज/काम आपकी मदद करेगा?

आपके ख्‍याल से, किसी प्रतिष्‍ठान में इंफेक्‍शन क्‍यों फैलता है? इसके लिए कौन जिम्‍मेदार है? डिलीवरी के दौरान, किस वक्‍त इंफेक्‍शन फैलते हैं? पहले, उसके दौरान या बाद में?

इंफेक्‍शन को फैलने से रोकने के लिए क्‍या किया जा सकता है? हाथ धोना? इंफेक्‍शन की रोकथाम करने का प्रोटोकॉल? बच्‍चा पैदा होने की जगह को साफ करना?

इंफेक्‍शन फैल जाने के बाद, उसके लक्षण दिखाई देने में कितना वक्‍त लगता है?

क्‍या आपको लगता है कि हाथ धोने के खराब नतीजे हो सकते हैं? क्‍या यह आपके साथ कभी हुआ है, क्‍या आप हमें ऐसी कोई कहानी सुना सकती हैं जो आपने शायद दूसरे प्रतिष्‍ठानों से सुनी हो?

इंफेक्‍शन की रोकथाम और उसका पता लगाने के बारे में समुदाय और मरीजों को कितनी जानकारी है? क्‍या आप उन्‍हें इस बारे में कुछ बताती हैं? स्‍वच्‍छता की अहमियत और इंफेक्‍शन के खतरे के बारे में सफाई करने वाले कर्मचारियों को कितनी जानकारी है? क्‍या आप उन्‍हें इस बारे में कुछ बताती हैं?

EXPERIENCED SET

**5.** सुनीता, पुष्‍पा और मीना तीन अलग-अलग ब्‍लॉकों के CHCs में तीन स्‍टाफ नर्स हैं। वे सीनियर स्‍टाफ नर्सें हैं और अपने बीच उन अनुभवों पर सामान्‍य बातचीत कर रही हैं जो उनके अपनी जूनियर स्‍टाफ नर्सों के साथ रहे हैं:

**5.1** उन्‍होंने पाया है कि अक्‍सर जूनियर नर्स थोड़े एनीमिया की जरूरत रखने वाले केसिस को ज़िला अस्‍पताल में रेफर कर देती हैं। वे इसके लिए अपने अस्‍पतालों में विभिन्‍न हालातों को जिम्‍मेदार ठहराती हैं:

A. सुनीता के अस्‍पताल में ज़ि‍ले के अफसर जांच करने के लिए लगातार आते रहते हैं, अस्‍पताल के कार्य की जवाबदेही अक्‍सर उस पर आ जाती है। बुरे परिणामों के लिए उसे अपने सीनियर्स की अक्‍सर डांट खानी पड़ती है।

B. पुष्‍पा के अस्‍पताल में वहां आने वाले मरीजों की संख्‍या की तुलना में स्‍टाफ कम है। इसलिए स्‍टाफ पर काम का बोझ बहुत ज्‍यादा है और अक्‍सर दबाव में रहते हैं।

C. मीना के अस्‍पताल में, स्‍टाफ नर्सों का LMO के साथ अच्‍छा कामकाजी संबंध और बातचीत नहीं है। नर्सें HRPs और मुश्किलों को संभालने में मदद के लिए LMO के पास जाना नहीं चाहती हैं।

कौन सी जूनियर स्‍टाफ नर्स एन‍ीमिया वाले केसिस को ज़िला अस्‍पताल में रेफर किए जाने की सबसे ज्‍यादा संभावना रखती है?

- गर्भवती महिला को प्रतिष्‍ठान में भर्ती करने का क्‍या प्रोटोकॉल है? वह पहले कहां जाती है?

अपने प्रतिष्‍ठान में एक ठेठ प्रसव-पूर्व जांच के बारे में हमें बताइये।

- क्‍या? कौन-कौन से पैरामीटरों की जांच की जाती है? मेडिकल हिस्‍ट्री?

- कहां? क्‍या यह जगह बदलती रहती है या एक फिक्‍स्‍ड जगह है?

- उसके महत्‍वपूर्ण अंगों की जांच कौन करता है? कौन इंचार्ज है?

- अगर कोई कॉम्‍प्‍लीकैशन है तो मरीज को रेफर करने का फैसला कौन करता है?

- यह फैसला कैसे किया जाता है? यह फैसला करने के लिए कौन सा क्राइटेरिया इस्‍तेमाल किया जाता है?

- मरीज को बाहर रेफर करने से पहले, कौन से इंतजाम करने होते हैं?

- कहां और कौन से वाइटल्‍स रिकॉर्ड किए जाते हैं? किसके द्वारा? कब और कैसे आप डिलीवरी के दौरान इन रिकॉर्डों को इस्‍तेमाल करती हैं?

- प्रसव-पूर्व जांचों को करने और HRPs को रेफर करने में आपको किस तरह की दिक्‍कतों का सामना करना पड़ता है? आप इन दिक्‍कतों पर कैसे काबू पाती हैं? उदाहरण बताइये।

- क्‍या वहां ऐसे केस हुए हैं जब रेफर किए गए मरीज को DH पहुंचने से पहले गंभीर कॉम्‍प्‍लीकैशनों का सामना करना पड़ा हो? आपके ख्‍याल से, किस वजह से कॉम्‍प्‍लीकैशन पैदा होती है? उससे कैसे बचा जा सकता था? क्‍या आपने कुछ अलग तरीके से किया होता?
- क्‍या आपके प्रतिष्‍ठान में भारी वर्कलोड है? वर्कलोड से रेफरल और उससे संबंधित निर्णय लेने के प्रोसेस पर कैसे असर पड़ता है?

रेफरल प्रोसेस और निर्णय में LMO से किस तरह के तालमेल की जरूरत होती है? क्‍या वहां कोई फिक्‍स्‍ड प्रोटोकॉल है? इस काम में और कौन-कौन शामिल होता/मदद करता है? क्‍या आपको तालमेल बिठाने में कभी कोई दिक्‍कत हुई है? आप इन दिक्‍कतों पर कैसे काबू पाती हैं? LMO/मदद मौजूद नहीं होने पर आप क्‍या करते हैं? कोई उदाहरण बताइये जब आपने रेफरल/काम को संभालने के लिए LMO से मदद मांगी हो?

**5.2** वे बातचीत कर रही हैं कि उनके अस्‍पतालों में 48 घंटे तक रुकने वाले केसिस की संख्‍या इतनी कम क्‍यों है।

A. सुनीता का कहना है कि महिलाएं इस कारण से चली जाती हैं क्‍योंकि उनकी ASHAs (बहुएं) चली जाती हैं, और वे महिला के फैसले को सबसे ज्‍यादा प्रभावित करती हैं।

B. पुष्‍पा का कहना है कि महिलाएं इस कारण से जाती हैं क्‍योंकि स्‍टाफ के पास मरीज को मॉनीटर और अटैंड करने के लिए काफी समय नहीं है।

C. मीना का कहना है कि महिलाएं इस कारण से चली जाती हैं क्‍योंकि अस्‍पताल, लेबर वार्ड और दूसरी सुविधाओं की हालत खराब है, बिल्‍कुल भी सुविधाजनक या आरामदायक नहीं है।

किस स्‍टाफ नर्स के द्वारा अस्‍पताल में डिलीवरी करवाने के बाद ज्‍यादातर महिलाओं के चले जाने की सबसे ज्‍यादा संभावना है?

क्‍या आपके ख्‍याल से, महिलाओं के लिए 48 घंटे या 24 घंटे रुकना जरूरी है? महिलाओं और परिवारों को प्रतिष्‍ठान में ज्‍यादा वक्‍त तक रुकवाने के लिए, आप क्‍या कर सकती हैं? आपने क्‍या-क्‍या कोशिश की है, क्‍या कामयाब हुआ है? क्‍या कामयाब नहीं हुआ है? अपने तरीके को बदलने के लिए, आपने क्‍या किया था?

डिलीवरी होने के बाद, परिवारों द्वारा जल्‍दबाजी किए जाने के लिए कौन से मुख्‍य कारण हैं? कौन सबसे ज्‍यादा जोर डालता है – महिलाएं, रिश्‍तेदार, ASHA?

उन परिवारों से आपको किस तरह की शिकायतें मिली हैं जो रुकना चाहते थे, लेकिन उनका अनुभव अच्‍छा नहीं रहा?

डिलीवरी के बाद, पहले 2-48 घंटे में किस तरह के कॉम्‍प्‍लीकैशन्‍स और खतरे पैदा हो सकते हैं? क्‍या आप इस बारे में महिलाओं/परिवार वालों को बताती हैं? क्‍या वे इसे समझते हैं? क्‍या ASHA को इन कॉम्‍प्‍लीकैशन्‍स की जानकारी है?

**5.3** उन्‍हें अपने अस्‍पतालों में काम करते हुए काफी लंबा अर्सा हो गया है, और उन्‍होंने अपने अस्‍पताल और गांवों में अपनी-अपनी अच्‍छी-खासी इज्‍जत बना ली है। वे अपनी जूनियर स्‍टाफ नर्सों को निम्‍नलिखित सलाह देती हैं:

A. सुनीता अपनी जूनियर को जल्‍दी से डिलीवरी करवाकर नाम कमाने की सलाह देती है।

B. पुष्‍पा का विश्‍वास है कि मुश्किल और रिस्‍की डिलीवरी केसिस को संभाल कर इज्‍जत कमाई जा सकती है।

C. मीना का सोचना है कि अपने इलाके की ASHAs के बीच अच्‍छी इज्‍जत होने से समाज में भी उनकी इज्‍जत बढ़ेगी।

ज्‍यादातर मरीजों द्वारा किसकी सलाह माने जाने की संभावना है?

क्‍या मरीजों की मांगों को एक नर्स या दूसरी नर्स ने संभालना होता है? किस क्राइटेरिया के आधार पर, वे यह निर्णय लेती हैं? इसमें ASHAs क्‍या भूमिका निभाती हैं? क्‍या उनकी कोई प्रीफरेंस होती है?

किस वक्‍त महिलाओं को ऑक्‍सीटोसिन दिया जाना चाहिए, और किस वजह से?

हमने यह भी सुना है कि कभी-कभी ऑक्‍सीटोसिन इसलिए दिया जाता है क्‍योंकि महिला जल्‍दी डिलीवरी चाहती है? क्‍या आपके यहां भी यह होता है? क्‍या आपने अपनी साथी नर्सों यह बर्ताव करते हुए देखा है? आपके ख्‍याल से, वे यह बर्ताव क्‍यों करती हैं?

क्‍या आपको लगता है कि नर्सें जल्‍दी डिलीवरी चाहती हैं? इससे नर्सों को कैसे फायदा होता है? हमने यह भी सुना है कि ऑक्‍सीटोसिन के इस्‍तेमाल के बाद नर्सों को किसी तरह की रिश्‍वत मिलती है – क्‍या यह एक कारण है जिसकी वजह से लोग गलत तरीके से ऑक्‍सीटोसिन इस्‍तेमाल करते हैं?

**5.4** प्री-टर्म बर्थ को संभाले के लिए दूसरी नर्सों को ट्रेनिंग देने के लिए उन्‍हें मास्‍टर ट्रेनर्स के रूप में ट्रेनिंग दी गई थी। ट्रेनिंग के दौरान, उनमें से हर-किसी प्री-टर्म बर्थ के खतरे बताने का अपना एक खास तरीका था।

A. सुनीता राज्‍य और देश में प्री-टर्म डिलीवरी में बच्‍चों की मौत की अत्‍यधि उच्‍च संख्‍या के बारे में बात करती है। वह यह बात समझाने की कोशिश करती है कि नवजन्‍मे बच्‍चों की मौत के लिए प्री-टर्म डिलीवरी सबसे बड़ा कारण है।

B. पुष्‍पा प्री-टर्म डिलीवरी के कारण नवजन्‍मे बच्‍चों की मौत बार-बार और बहुत बार होने के बारे में बताने की कोशिश करती है। वो बताती है कि अगर कोई अस्‍पताल एक साल में 1000 बच्‍चों की डिलीवरी करवाता है तो वहां औसत हर महीने प्री-टर्म डिलीवरी के कारण एक मौत होगी।

C. मीना प्री-टर्म डिलीवरी के कारण मुश्किलों और मौतों से जुड़े अपने अनुभवों के बारे में बात करती है। वो प्री-टर्म केस में अपनी असफलता के कारण कुछ मौतों के बारे में बताती है।

इन तीन स्‍टाफ नर्सों में किसके द्वारा ट्रेनिंग के दौरान प्री-टर्म डिलीवरी के खतरे सफलतापूर्वक सूचित किए जाने की सबसे ज्‍यादा संभावना है?

आपके पास एक हफ्ते में कितने HRP केसिस आते हैं? उनमें से कितने मॉडरेट और कितने हाई रिस्‍क होते हैं? आप कौन से केसिस को सबसे ज्‍यादा देखती हैं – प्रीमैच्‍योरिटी, एनीमिया, ईक्‍लैम्‍पसिया? आप किस तरह के HRPs को सबसे ज्‍यादा रेफर करती हैं? आप किस HRP कंडीशन को आराम से संभाल सकती हैं?
